# Heterogeneity of Estimated GFR Slopes According to Etiology, Estimated GFR and Urinary Albumin-to-Creatinine Ratio in a Large Cohort of Patients With CKD

**DOI:** 10.1016/j.ekir.2025.11.021

**Published:** 2025-11-20

**Authors:** Charlotte Behning, Ulla T. Schultheiss, Jennifer Nadal, Heike Meiselbach, Sebastian Schönherr, Lukas Forer, Elke Schaeffner, Vera Krane, Markus P. Schneider, Matthias Schmid, Florian Kronenberg, Anna Köttgen, Kai-Uwe Eckardt, Fruzsina Kotsis, GCKD study investigators, Kai-Uwe Eckardt, Heike Meiselbach, Markus P. Schneider, Mario Schiffer, Hans-Ulrich Prokosch, Barbara Bärthlein, Andreas Beck, André Reis, Arif B. Ekici, Susanne Becker, Ulrike Alberth-Schmidt, Anke Weigel, Sabine Marschall, Eugenia Schefler, Gerd Walz, Anna Köttgen, Ulla T. Schultheiß, Fruzsina Kotsis, Simone Meder, Erna Mitsch, Ursula Reinhard, Jürgen Floege, Turgay Saritas, Alice Groß Charité, Elke Schaeffner, Seema Baid-Agrawal, Kerstin Theisen, Kai Schmidt-Ott, Martin Zeier, Claudia Sommerer, Mehtap Aykac, Gunter Wolf, Martin Busch, Andy Steiner, Thomas Sitter, Christoph Wanner, Vera Krane, Antje Börner-Klein, Britta Bauer, Florian Kronenberg, Julia Raschenberger, Barbara Kollerits, Lukas Forer, Sebastian Schönherr, Hansi Weissensteiner, Peter Oefner, Wolfram Gronwald, Matthias Schmid, Jennifer Nadal

**Affiliations:** 1 Department of Medical Biometry, Informatics, and Epidemiology, University Hospital Bonn, Bonn, Germany; 2 Faculty of Medicine and Medical Center, Institute of Epidemiology and Prevention, University of Freiburg, Freiburg, Germany; 3 Department of Medicine IV, Nephrology and Primary Care, Faculty of Medicine and Medical Center, University of Freiburg, Freiburg, Germany; 4 Department of Nephrology and Hypertension, Friedrich-Alexander-Universität Erlangen-Nürnberg, Erlangen, Germany; 5 Institute of Genetic Epidemiology, Medical University of Innsbruck, Innsbruck, Austria; 6 Institute of Public Health, Charité—Universitätsmedizin Berlin, Berlin, Germany; 7 Department of Clinical Research and Epidemiology, German Heart Failure Center, University Hospital Würzburg, Würzburg, Germany; 8 Division of Nephrology, Department of Medicine I, University Hospital Würzburg, Würzburg, Germany; 9 Department of Nephrology and Medical Intensive Care, Charité—Universitätsmedizin Berlin, Berlin, Germany

**Keywords:** CKD progression, eGFR slope, etiology, heterogeneity

## Abstract

**Introduction:**

The rate of decline in estimated glomerular filtration rate (GFR, eGFR) is increasingly recognized as a quantitative marker of chronic kidney disease (CKD) progression. However, data on eGFR slopes have mainly been reported in cohorts enriched for fast progression and the heterogeneity of eGFR slopes across the spectrum of CKD remains poorly defined.

**Methods:**

In 5214 participants of the German CKD (GCKD) study, we modeled eGFR slopes using per-protocol and clinical measurements. We used linear-mixed effects models, with eGFR slope as the outcome and baseline demographics as independent variables to (i) describe eGFR slope heterogeneity; (ii) assess differences by CKD etiology, eGFR and urinary albumin-to-creatinine ratio (UACR) categories, sex, and age; and (iii) determine associations of slopes with estimated eGFR decline (30%, 40%, and 57%) and observed end points (kidney failure with replacement therapy, mortality).

**Results:**

On average, 9 eGFR values per participant (interquartile range: 7–12) over 6.5 years were used for slope calculation. The adjusted mean annual eGFR slope was −1.43 ml/min per 1.73 m^2^. Slopes were similar across eGFR categories, but steeper with higher UACR. Faster eGFR decline was observed in participants of younger age and in those with polycystic kidney disease or diabetic kidney disease (DKD). Although eGFR slopes did not consistently differ by sex, women with diabetes as the leading cause of CKD had lower slopes than their male counterparts. A rapid annual decline (> 5 ml/min per 1.73 m^2^) occurred in 4.3%, with variation in frequency by CKD cause and UACR.

**Conclusion:**

In conclusion, though the average eGFR slope was low, it varied considerably, depending on CKD etiology and UACR. This data may help to put slope estimates in individual patients and defined subpopulations into perspective.

## Graphical abstract

**Figure:**
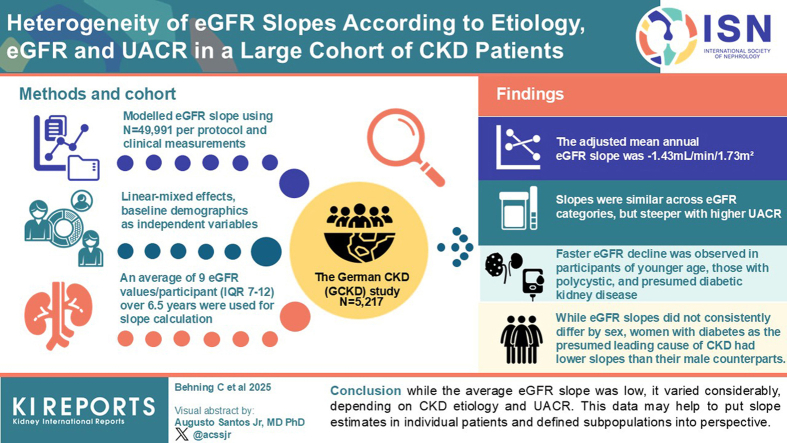

CKD represents a major global health burden because of its worldwide prevalence of > 10% and its severe associated risks, including kidney failure treated with kidney replacement therapy (KFRT), cardiovascular events, and premature death.^1^ However, the course of CKD is highly variable, ranging from stable to rapidly progressive disease. A deeper understanding of CKD heterogeneity is required for improved risk stratification and targeted therapies.

Rates of KFRT and death have traditionally been employed as “hard end points” to assess CKD heterogeneity. Large meta-analyses showed that the relative loss of eGFR by 30%, 40%, and 57% is associated with the subsequent development of KFRT and death.^2^ These observations highlighted the potential of change in GFR as an indicator for CKD progression, thereby facilitating the design of clinical trials.^3^^,^^4^ More recently, the annual rate of decline of eGFR, also designated as “eGFR slope,” has received increasing attention as a quantitative measure of CKD progression.^5^, ^6^, ^7^ In fact, change in eGFR slopes has been accepted as a clinical end point for the evaluation and approval of CKD therapies.^8^ Compared with dichotomous end points, eGFR slope as a continuous variable has the advantage that it can be determined for each individual, irrespective of whether a specific GFR end point has been met, and reflects CKD heterogeneity at much higher granularity. However, a sufficient number of creatinine measurements is necessary to calculate slopes. Furthermore, it is crucial to differentiate between the decline in eGFR due to actual progression of CKD and the natural age-related decline in healthy individuals, estimated at approximately 0.8 to 1.0 ml/min per 1.73 m^2^/yr in adults.^9^, ^10^, ^11^

Although early studies suggested that CKD progression is characterized by a steady, linear decline in eGFR,^12^ subsequent investigations revealed significant heterogeneity of eGFR trajectories.^13^ Some patients exhibit approximately linear declines but with varying slopes across individuals,^14^, ^15^, ^16^ whereas others show nonlinear declines over time.^17^^,^^18^ Associations of these eGFR slope patterns with KFRT and other adverse events have been observed.^19^^,^^20^ However, most data were reported from cohorts intentionally enriched for rapid CKD progressors.^21^ The sparseness of broader studies of sufficient duration and density of creatinine measurements has resulted in a lack of clarity regarding the associations and heterogeneity of eGFR slopes.^22^ The recent update of the Kidney Disease: Improving Global Outcomes (KDIGO) CKD guidelines mentions eGFR slopes only in the context of clinical trial results, but not in the context of CKD assessment, stratification, or prognostication, thereby reflecting limited evidence.^23^ “Rapid” CKD progression has been defined as an annual eGFR decline > 5 ml/min per 1.73 m^2 23^; however, the proportion of patients with CKD with different characteristics that fulfills this criterion is unknown.

This study aimed to describe the heterogeneity of eGFR slopes across a wide spectrum of CKD in > 5000 individuals enrolled in the GCKD study, one of the world’s largest CKD cohort studies.^24^ Employing nearly 50,000 eGFR values from per-protocol measurements and routine clinical data over a 6.5-year follow-up period, we analyzed eGFR slopes across the CKD classification based on cause, eGFR, and albuminuria,^25^ as well as other participant characteristics. In addition to the description of the heterogeneity of eGFR slopes, we described their associations with estimated (relative loss of eGFR of 30%, 40%, or 57%) and observed end points (KFRT, mortality).

## Methods

### Study Population and Inclusion Criteria

The GCKD study enrolled 5217 participants between 2010 and 2012 with the following: (i) eGFR between 30 and 60 ml/min per 1.73 m^2^ (CKD G3, A1–3; *n* = 4774, 91.6%) or (ii) eGFR > 60 ml/min per 1.73 m^2^ and “overt” albuminuria (UACR ≥3 00 mg/g) (CKD G1–2, A3; *n* = 440, 8.4%). The mean eGFR at baseline was 47 ± 17 ml/min per 1.73m^2^ and the median UACR of 51 (interquartile range: 9–392) mg/g.^24^ According to the KDIGO risk categorization^19^ defined by GFR and UACR categories, 6.2%, 24.3%, 32.7%, and 36.7% were in low, moderate, high, and very high risk categories, respectively.^24^ Further details of the study design have been described previously.^26^^,^^27^ The following 5 main etiology categories (presumed leading cause of CKD) were considered for the current analysis based on judgement of the treating nephrologist at baseline: (i) autosomal dominant polycystic kidney disease (ADPKD), (ii) DKD, (iii) primary glomerular disease (PGD), (iv) hypertensive kidney disease (HKD), and (v) other kidney diseases (MISC). The latter included other systemic or hereditary diseases, interstitial kidney diseases, CKD following acute kidney injury, obstructive nephropathy, miscellaneous, and unknown causes.

### Statistical Methods

Details on data collection and measurements can be found in Supplementary Material S1. eGFR was estimated using the CKD-Epidemiology Collaboration 2009 equation as per study protocol and because the study population is ethnically homogenous of Caucasian origin.^24^ Any eGFR values collected after KFRT or during acute kidney injury (± 7 days of the acute event) were excluded. Acute kidney injury events were adjudicated by trained physicians from discharge reports and categorized based on the KDIGO definition^28^ and the GCKD study event adjudication catalog.^27^ The cleaned data set comprised data from 5214 participants for a period of ≤ 6.5 years following the baseline measurement (data freeze: March, 2022; Supplementary Figure S1). All analyses were performed in R, version 4.2.2 (R Core Team, Vienna, Austria). Linear mixed-effects models were used to calculate individual eGFR slopes (Supplementary Material S2). Repeated measurements of a participant were included by random intercept and random slope term (= random effects).^29^^,^^30^ By modeling with linear mixed-effects models, it was possible to include participants with only 1 eGFR value during the study period to estimate the eGFR decline at the population level. In a basic reference model 1, no other independent variables were included besides the follow-up time in years and the random effects. In an adjusted model 2, the baseline variables UACR, sex, age, and disease etiology were additionally included as fixed effects and as interaction effects with the follow-up time using a complete case dataset. Based on model 2, eGFR slopes were stratified by slope quintiles, by disease etiology, and by categories of eGFR (G1–G5) and albuminuria (A1–A3). In addition, separately for each disease etiology, we examined the effect of the interaction of sex and age category (older than median vs. younger) as a fixed effect and in interaction with the follow-up time (model 3 A–E, Supplementary Material S2). All analyses described above were performed using different sources of serum creatinine estimates (Supplementary Figure S1). Additional sensitivity analyses for model 1 and model 2 were performed using only the follow-up visit values. To give a descriptive analysis of the heterogeneity of the eGFR slopes, we stratified some results in quintiles. This may aid in understanding the distribution of data, identify subgroups of decline and support meaningful comparisons. Unless otherwise stated, slopes are given in ml/min per 1.73 m^2^/yr.

### Estimated and Observed End Points

We described the associations of eGFR slopes with estimated and observed end points (EP). Estimated end points were as follows: 30%, 40%, and 57% eGFR decline, whereby each participant's modeled eGFR (model 2) was assessed for whether it fell below 30%, 40%, or 57% of the baseline eGFR value (Supplementary Figure S2), and the estimated time at which the end points was reached was inferred. Observed end points were as follows: (i) KFRT (dialysis or transplantation), (ii) kidney death (death because of forgoing of dialysis), and (iii) all-cause death. In this paper, we focused on eGFR slope heterogeneity based on slope modelling, end points, and descriptive strategy, and do not apply survival methodology. Using our results, future analyses may focus on time to event analyses with the estimated slopes and derived end points. The eGFR slopes derived from the model comprised both covariate-dependent and participant-specific slopes. Estimated end points were not reported for participants with missing baseline eGFR data.

## Results

### Size and Density of the Data Set

As illustrated in Supplementary Figure S1, a total of v = 81,218 eGFR values from 5217 study participants were exported. After data cleaning, which comprised the exclusion of eGFR values obtained after KFRT (v = 4076), or during acute kidney injury events (v = 2530), censoring for 6.5 years (v = 19,732), and removal of 3 participants lacking any eGFR values, 49,991 values from 5214 participants remained for further analyses. Thereof, v = 15,186 (30%) values were derived from in-person follow-up study visits and v = 34,805 (70%) from medical reports. The median number of eGFR values per participant was 9 (interquartile range: 7–12) (Supplementary Table S2). Ninety-two participants lacking baseline UACR values were excluded from model 2 (Supplementary Table S1).

### Baseline Characteristics

Baseline participant characteristics varied by CKD etiology, eGFR, and UACR category (Supplementary Tables S2–S4). The distribution of kidney disease etiologies was as follows: HKD (1198, 23.0%), PGD (977, 18.7%), DKD (783, 15.0%), ADPKD (191, 3.7%), and MISC (2065, 39.6%). The mean age at baseline was 60.1 ± 12.0 years, lowest in the PGD group (52.7 ± 13.6 years) and highest in the HKD (64.7 ± 8.5 years) and DKD (64.5 ± 8.0 years) groups. Overall, 60% of participants were men, with the highest proportion in the DKD group (68.5%) and balanced sex ratios in the MISC and ADPKD groups (Supplementary Table S2).

Comparison of participants according to eGFR categories revealed that those in stages G1 or G2 were younger and, consistent with study inclusion criteria, had higher UACR values than those in higher G stages (Supplementary Table S3). Stratification by baseline UACR values revealed that those with a UACR < 30 mg/g were older and more likely to have been diagnosed with HKD, whereas participants with UACR > 300 mg/g were younger, more likely to be male and more frequently diagnosed with PGD (Supplementary Table S4).

### Characteristics and Heterogeneity of eGFR Slopes

To assess the heterogeneity of eGFR slopes, we stratified our data by quintiles of covariate-adjusted, annual eGFR decline (Q1–5: fast, fast-medium, medium, slow-medium, and slow) of the modeled eGFR slope (Table 1). Participants in the fast quintile (between −14.5 and −2.76 ml/min per 1.73 m^2^/yr) were younger, more likely to be male, and had higher baseline UACR values. Most prevalent disease etiologies in the fast quintile were PGD (248, 24.2%) and DKD (227, 22.1%). Participants in the slow quintile (between −0.07 and +6.53 ml/min per 1.73 m^2^/yr) were more likely to be female and diagnosed with HKD (288, 28.1%). Majority of participants with ADPKD, DKD, or PGD were observed in the fast or fast-medium quintiles (Q1 + 2: 88.0%, 51.21%, 8.9%, respectively), whereas the majority of HKD and MISC participants were in the slow-medium or slow quintiles (Q4 + 5: 593, 49.50%, 946, 45.81%, respectively). When stratifying the overall group by eGFR categories at baseline, slopes were relatively similar, with participants with G3a progressing the slowest (annual slope: −1.27) (Supplementary Table S5).

**Table 1:** Characteristics of participants stratified by quintiles of modeled eGFR decline according to the eGFR progression model including covariates

Characteristics of participants		Overall *N* = 5214	Fast [−14.5 to −2.76] *n* = 1025	Fast-medium [−2.76 to −1.63] *n* = 1024	Medium [−1.63 to −0.833] *n* = 1024	Slow-medium [−0.833 to −0.0698] *n* = 1024	Slow [−0.0698 to 6.53] *n* = 1025
Age (yrs)	Mean (SD)	60.1 (12.0)	56.3 (12.9)	59.8 (12.3)	61.9 (11.1)	61.7 (10.9)	60.6 (11.5)
Sex	male	3131 (60.0%)	692 (67.5%)	640 (62.5%)	599 (58.5%)	564 (55.1%)	584 (57.0%)
eGFR categories (ml/min per 1.73 m^2^)	G4+G5: < 30	504 (9.7%)	140 (13.7%)	114 (11.1%)	88 (8.6%)	73 (7.1%)	81 (7.9%)
	G3b: 30–44	1905 (36.5%)	374 (36.5%)	391 (38.2%)	402 (39.3%)	373 (36.4%)	337 (32.9%)
	G3a: 45–59	1713 (32.9%)	284 (27.7%)	309 (30.2%)	340 (33.2%)	368 (35.9%)	376 (36.7%)
	G2: 60–89	861 (16.5%)	174 (17.0%)	160 (15.6%)	149 (14.6%)	170 (16.6%)	192 (18.7%)
	G1: ≥ 90	231 (4.4%)	53 (5.2%)	50 (4.9%)	45 (4.4%)	40 (3.9%)	39 (3.8%)
UACR categories (mg/g)	< 30	2188 (42.0%)	139 (13.6%)	309 (30.2%)	445 (43.5%)	635 (62.0%)	660 (64.4%)
	(30–299)	1491 (28.6%)	218 (21.3%)	316 (30.9%)	400 (39.1%)	293 (28.6%)	264 (25.8%)
	(300–3000)	1297 (24.9%)	557 (54.3%)	389 (38.0%)	169 (16.5%)	89 (8.7%)	93 (9.1%)
	> 3000	146 (2.8%)	111 (10.8%)	10 (1.0%)	10 (1.0%)	7 (0.7%)	8 (0.8%)
	Missing	92 (1.8%)	0 (0%)	0 (0%)	0 (0%)	0 (0%)	0 (0%)
Inclusion criteria	G3, A1–A3	4774 (91.6%)	906 (88.4%)	923 (90.1%)	945 (92.3%)	957 (93.5%)	956 (93.3%)
	G1–2, A3	440 (8.4%)	119 (11.6%)	101 (9.9%)	79 (7.7%)	67 (6.5%)	69 (6.7%)
Med	ACEI	2471 (47.4%)	537 (52.4%)	509 (49.7%)	468 (45.7%)	436 (42.6%)	480 (46.8%)
	ARBs	2351 (45.1%)	529 (51.6%)	472 (46.1%)	454 (44.3%)	432 (42.2%)	424 (41.4%)
	Missing	35 (0.7%)	5 (0.5%)	4 (0.4%)	8 (0.8%)	8 (0.8%)	9 (0.9%)
Disease etiology	ADPKD	191 (3.7%)	131 (12.8%)	37 (3.6%)	12 (1.2%)	10 (1.0%)	0 (0%)
	Diabetic kidney disease	783 (15.0%)	227 (22.1%)	174 (17.0%)	174 (17.0%)	106 (10.4%)	78 (7.6%)
	Primary glomerular disease	977 (18.7%)	248 (24.2%)	230 (22.5%)	171 (16.7%)	142 (13.9%)	174 (17.0%)
	Hypertensive kidney disease	1198 (23.0%)	139 (13.6%)	198 (19.3%)	248 (24.2%)	305 (29.8%)	288 (28.1%)
	MISC	2065 (39.6%)	280 (27.3%)	385 (37.6%)	419 (40.9%)	461 (45.0%)	485 (47.3%)
Observed EPs	Kidney death	25 (0.5%)	15 (1.5%)	6 (0.6%)	2 (0.2%)	0 (0%)	1 (0.1%)
	KFRT	487 (9.3%)	356 (34.7%)	75 (7.3%)	32 (3.1%)	13 (1.3%)	5 (0.5%)
	All-cause death	679 (13.0%)	201 (19.6%)	149 (14.6%)	126 (12.3%)	100 (9.8%)	83 (8.1%)

ACEI, angiotensin-converting enzyme inhibitors; ADPKD, autosomal dominant polycystic kidney disease; ARB, angiotensin receptor blockers; eGFR, estimated glomerular filtration rate; EP, end point; KFRT, kidney replacement therapy; MISC, other kidney diseases; UACR urinary albumin-to-creatinine ratio.

eGFR categories, UACR categories, and age at baseline. eGFR categories (G1–G5) and eGFR are given in ml/min per 1.73 m^2^. Mean reported with ±SD, or as absolute and relative frequencies. Mean slope overall: −1.43 (1.86) and in quintiles: fast: −4.19 (1.42), fast-medium: −2.15 (0.33), medium: −1.20 (0.23), slow-medium: −0.48 (0.21), slow: 0.85 (0.94). Participants with missing baseline UACR are excluded from model 2 and thus from determining rapid decline. Characteristics of these participants can be found in Supplementary Table S1.

**Figure 1:**
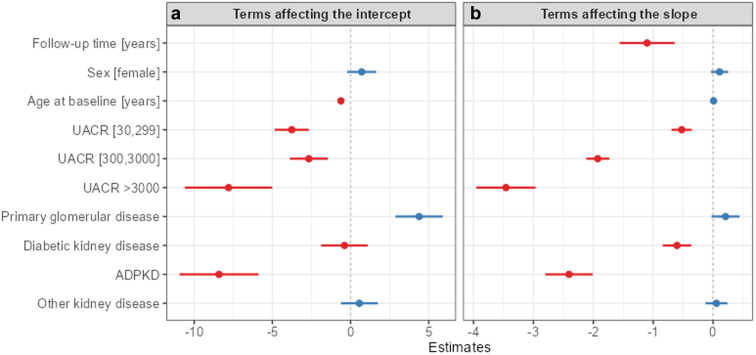
Forest plot of model coefficients that affect the intercept (baseline estimated glomerular filtration rate) and the estimated glomerular filtration rate slope. In model 2, the reference category includes male sex, UACR < 30 mg/g and as disease etiology hypertensive kidney disease. Panel A shows the main effects of the model whereas panel B shows the interaction effects with time. The model intercept ($\beta_{\;0}=87.33$, 95% confidence interval [84.39–90.27]) is not shown. See Supplementary Table S3 for further model details. Positive effects are marked with blue, negative with red. ADPKD, autosomal dominant polycystic kidney disease; UACR, urinary albumin-to-creatinine ratio.

Participants had an unadjusted overall mean annual eGFR slope of −1.38 (Supplementary Table S6). After adjusting for baseline covariates, the mean annual eGFR slope was −1.43 (Table 2) and varied considerably between the fastest (−4.19) and slowest (0.85) quintiles (Table 1). In model 2 (Figure 1, Supplementary Table S6), the coefficient for the follow-up time describes the change in eGFR over time for a participant belonging to the reference category of all covariates (male, UACR < 30, HKD etiology, theoretical age of 0 years). There was no difference in eGFR slope by sex (slope parameter, β = 0.11, 95% confidence interval (CI): −0.04 to 0.25). Compared with HKD, eGFR slopes did not differ in participants with MISC (β = 0.06, 95% CI: −0.13 to 0.24) or PGD (β = 0.21, 95% CI: −0.03 to 0.44), whereas participants with DKD (β = −0.60, 95% CI: −0.84 to −0.37) and ADPKD (β = −2.41, 95% CI: −2.80 to −2.01) had a faster eGFR decline. In addition, younger age (β = 0.01, 95% CI: 0.0016–0.0145) and higher UACR (β = −0.52, 95% CI: −0.69 to −0.36; β = −1.93, 95% CI: −2.12 to −1.73; β = −3.46, 95% CI: −3.95 to −2.96) at baseline were associated with a steeper annual decline of eGFR.

**Table 2:** Model-based eGFR slope, eGFR related and other end points stratified by disease etiology

eGFR slope and related end points		ADPKD (*n* = 190)	Diabetic kidney disease (*n* = 759)	Primary glomerular disease (*n* = 965)	Hypertensive kidney disease (*n* = 1178)	MISC (*n* = 2030)	Overall (*N* = 5122)
Model (2) slope	eGFR slope rate						
	Mean (SD)	−3.53 (1.60)	−1.98 (1.83)	−1.80 (2.14)	−0.96 (1.54)	−1.13 (1.71)	−1.43 (1.86)
	Median [Q25, Q75]	−3.54 (-4.64, −2.34)	−1.69 (-3.05, −0.86)	−1.61 (-2.83, −0.48)	−0.82 (-1.81, −0.09)	−0.93 (-2.01, −0.12)	−1.18 (-2.41, −0.31)
	Rapid decline	33 (17.4%)	52 (6.9%)	70 (7.3%)	17 (1.4%)	49 (2.4%)	221 (4.3%)
Estimated EPs derived from model (2)	Number and frequency reaching estimated EP: *n* (%)						
	30% eGFR	146 (76.8%)	322 (42.4%)	336 (34.8%)	280 (23.8%)	529 (26.1%)	1613 (31.5%)
	40% eGFR	125 (65.8%)	236 (31.1%)	244 (25.3%)	174 (14.8%)	322 (15.9%)	1094 (21.4%)
	57% eGFR	94 (49.5%)	142 (18.7%)	138 (14.3%)	68 (5.8%)	161 (7.9%)	603 (11.8%)
	Missing	0 (0%)	1 (0.1%)	0 (0%)	0 (0%)	1 (0.0%)	2 (0.0%)
	Time to EP: median (Q25, Q75)						
	30% eGFR	2.96 (2.10, 3.98)	3.54 (2.26, 4.76)	3.49 (2.22, 4.90)	4.15 (3.02, 5.25)	4.03 (2.73, 5.26)	3.73 (2.47, 5.03)
	40% eGFR	3.62 (2.76, 4.44)	3.99 (2.79, 5.31)	4.03 (2.78, 5.23)	4.65 (3.59, 5.49)	4.47 (3.15, 5.29)	4.24 (3.03, 5.27)
	57% eGFR	4.62 (3.52, 5.54)	4.63 (3.63, 5.62)	4.42 (3.59, 5.44)	5.30 (4.19, 5.98)	4.96 (3.89, 5.66)	4.76 (3.66, 5.65)
Observed EPs	Kidney death	1 (0.5%)	6 (0.8%)	5 (0.5%)	3 (0.3%)	9 (0.4%)	24 (0.5%)
	KFRT	60 (31.6%)	105 (13.8%)	122 (12.6%)	65 (5.5%)	129 (6.4%)	481 (9.4%)
	All-cause death	14 (7.4%)	189 (24.9%)	53 (5.5%)	176 (14.9%)	227 (11.2%)	659 (12.9%)

ADPKD, autosomal dominant polycystic kidney disease; eGFR, estimated glomerular filtration rate; EP, end point; KFRT, kidney failure treated by kidney replacement therapy (dialysis or transplantation); MISC, other kidney diseases; UACR urinary albumin-to-creatinine ratio.

Participants are stratified by disease etiology. For each participant, it is checked whether the modelled eGFR line falls below 30% (40%, 57%) of the baseline eGFR value (according to model 2). If this EP is reached (EP: 30%, 40%, 57% eGFR decline), it is also reported after which time after baseline (in yrs) the threshold is theoretically reached. The end point cannot be reported in 2 cases because of missing baseline eGFR. The slope terms presented in this table reflect added fixed and patient-specific slopes. For participants with only 1 observation, the slope resembles the fixed effects of the model for the given baseline parameters. The subpopulation with existing UACR baseline values (*N* = 5122) as used in model 2 is shown. eGFR slope rates are given in ml/min per 1.73 m^2^/yr. Negative values reflect decline. Rapid decline: is defined as decline of > 5 ml/min per 1.73 m^2^/yr. The mean annual rate of decline in participants with a rapid eGFR decline was −6.28. Kidney death: death because of forgoing of dialysis. Mean reported with ±SD, median with 25% and 75% quartiles (interquartile range: Q25, Q75) or as absolute and relative frequencies. The overall number of participants is based on the complete case dataset.

Sensitivity analyses, including only serum creatinine values obtained at follow-up visits resulted in comparable model results (Supplementary Table S7). Because the number of observations available were lower (median number of observations using only follow-up values: 3 compared with 10, including all values; Supplementary Tables S2–S4) and model fits resulted in convergence warnings, the following analyses focus on the model including eGFR values from all available sources.

### Differentiation of eGFR Slopes by Age, Sex, and Disease Etiology

To illustrate eGFR slopes by CKD etiology, we averaged mean eGFR across participants within 6-month-time windows (Supplementary Figure S3). To support the graphical conclusions, the results from model 3 are provided in Supplementary Table S8. Participants younger than the median age exhibited higher baseline eGFR values compared with older participants, except for the subgroup with ADPKD (Supplementary Table S8). Older participants with ADPKD, DKD, and PGD had a slower rate of eGFR decline (Table S8). Although no sex differences were observed overall (model 2), sex differences were evident in some subgroups in the separate models for each disease etiology (models 3 A–E, Supplementary Table S8). Participants with PGD and MISC showed higher baseline eGFR values for female participants in the younger subgroup (β = 5.26, 95% CI: 1.72–8.81 and β = 5.35, 95% CI: 3.34–7.36, respectively). For participants with DKD and MISC, the rate of eGFR decline was slower for females than males (β = 1.03, 95% CI: 0.29–1.76 and β = 0.32, 95% CI: 0.01–0.63, respectively), although these results may be affected by the different group sizes.

**Figure 2:**
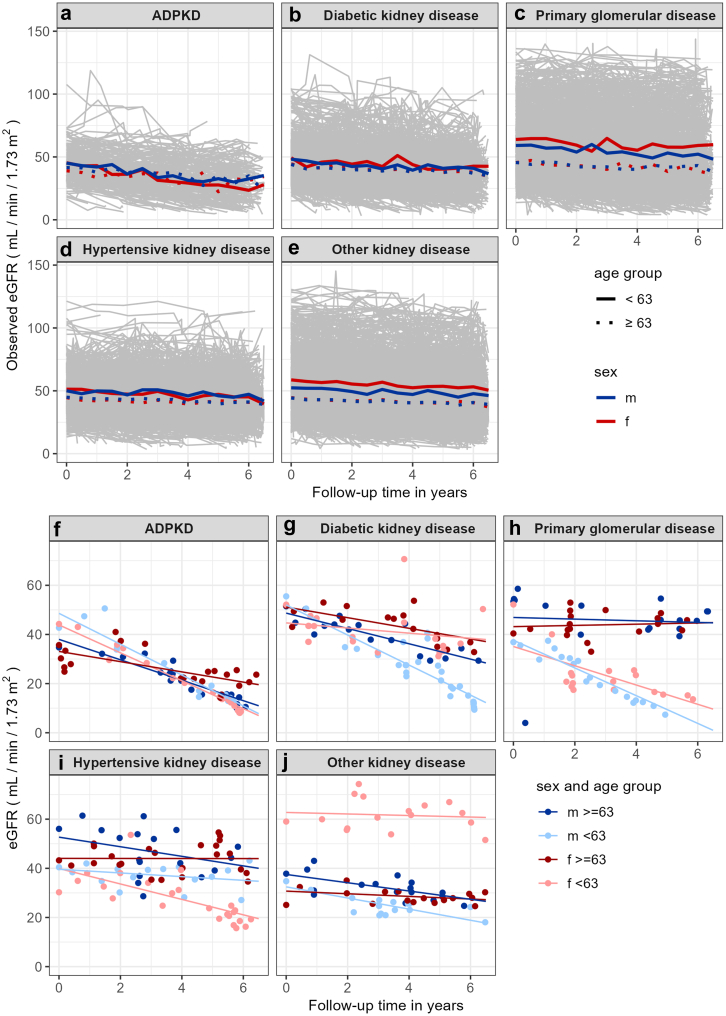
Modelled individual and mean eGFR slopes by disease etiology, age, and sex over 6.5 years follow-up. Modelled eGFR values as derived from model 3 for all participants after 6.5 years of follow-up by disease etiology, sex, and age group. Age subgroups are separated at the median age of 63 years. Upper panels (a–e): modelled eGFR slope for each participant (thin grey lines in the background) within the different disease etiology subgroups. Thick lines represent mean slopes within each disease etiology for age (solid line: < 63 years vs. dotted lines ≥ 63 years) and for sex (male: blue, red: female). Modelled eGFR values < 0 are treated as missing. Lower panels (f–j): Lines show individual examples of modelled eGFR slopes with the observed values (dots) for each participant within the different disease etiology subgroups. ADPKD, autosomal dominant polycystic kidney disease, eGFR: estimated glomerular filtration rate; f, female; m, male.

**Figure 3:**
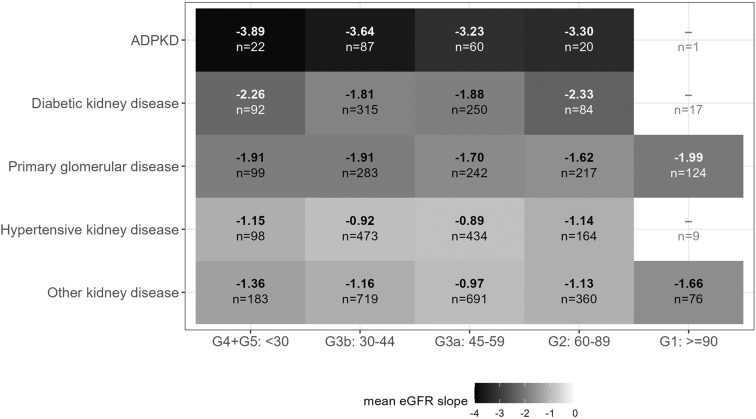
eGFR slope according to baseline eGFR categories in each disease etiology group. Mean eGFR slope rates in ml/min per 1.73 m^2^/yr for each disease etiology group (y-axis) and for different chronic kidney disease stages at baseline (x-axis) for > 20 participants (using model 2). According to the Kidney Disease: Improving Global Outcomes guidelines: CKD stage G1, ≥ 90 ml/min per 1.73 m^2^; stage G2, 60–89 ml/min per 1.73 m^2^; stage G3a, 45–59 ml/min per 1.73 m^2^; stage G3b, 30–44 ml/min per 1.73 m^2^; stage G4, 15–29 ml/min per 1.73 m^2^; and stage G5, < 15 ml/min per 1.73 m^2^ without kidney replacement therapy. ADPKD, autosomal dominant polycystic kidney disease; eGFR, estimated glomerular filtration rate.

**Figure 4:**
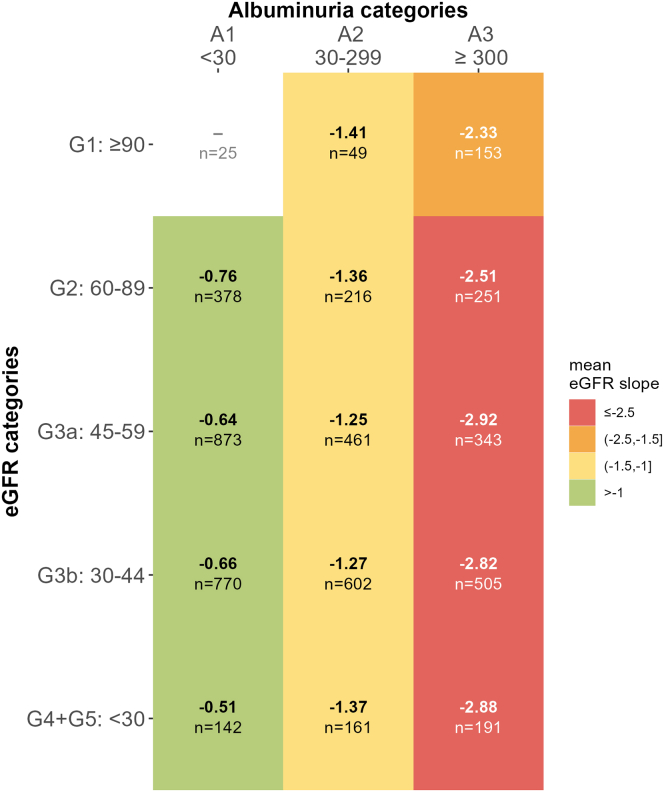
Modelled eGFR slope according to eGFR and albuminuria categories at baseline. Mean modeled eGFR slope in ml/min per 1.73 m^2^/yr for each eGFR (y-axis) and albuminuria (x-axis) category at baseline for > 30 participants (using model 2). The mean slope by albuminuria was A1: −0.66, A2: −1.29, A3: −2.75. According to Kidney Disease: Improving Global Outcomes guideline: CKD stage G1 ≥ 90 ml/min per 1.73 m^2^, stage G2 60–89 ml/min per 1.73 m^2^, stage G3a 45–59 ml/min per 1.73 m^2^, stage G3b 30–44 ml/min per 1.73 m^2^, stage G4 15–29 ml/min per 1.73 m^2^, and stage G5 < 15 ml/min per 1.73 m^2^ without kidney replacement therapy. Albuminuria expressed as urine albumin creatinine ratio in mg/g according to Kidney Disease: Improving Global Outcomes categories. eGFR, estimated glomerular filtration rate.

Both observed (Supplementary Figure S3) and modeled eGFR slopes (Figure 2, top panel) revealed heterogeneity within and overlap between disease etiologies. The heterogeneity of modeled slopes is further illustrated by example trajectories (Figure 2, bottom panel). Stratification by baseline eGFR category within each etiology group (Figure 3) also revealed large differences between etiologies and much smaller, inconsistent differences between eGFR categories. The fastest rate of eGFR decline was observed for ADPKD with G4/G5 (−3.9). For DKD participants with stages G4/G5 and G2, annual decline was the fastest (−2.3). In contrast, among those with PGD stage G1, the annual decline was fastest (−2.0). In participants with HKD, those with CKD stages G4/5 showed the fastest decline. Interestingly, for the whole cohort slopes were relatively similar across G1-G5 categories (Figure 4), whereas increasing albuminuria was directly proportional to faster eGFR decline (A1: −0.51 to −0.76, A2: −1.25 to −1.41, and A3: −2.33 to 2.92).

### Comparison of eGFR Slopes and End Points by Disease Etiology and UACR

As anticipated, the annual decline in eGFR was associated with the proportion of participants experiencing a predefined % reduction in eGFR. In the ADPKD subgroup which had the fastest mean annual eGFR decline (−3.53), a 40% eGFR decline was observed in 65.8% of participants after a mean follow-up time of 3.6 years (Table 2). In contrast, among participants with DKD and PGD, who had a fast-medium annual eGFR decline (−1.98 and −1.80, respectively), an eGFR decline of 40% occurred in 31.1% and 25.3% after approximately 4 years, respectively. Among participants with HKD and MISC with a mean eGFR decline of −0.96 and −1.13 the proportion of those reaching a 40% decline was only 14.8% and 15.9% after 4.7 and 4.5 years, respectively.

Similarly, KFRT, which occurred in 487 (9.3%) of all participants (Supplementary Table S9), was most frequent in the ADPKD (31.6%) and DKD (13.8%) groups, and least frequent in the HKD (5.5%) group (Table 2). KFRT rates were markedly different between the fast and slow quintiles of eGFR decline (34.7% vs. 0.5%) (Table 1).

A greater proportion of participants with a UACR > 3000 mg/g reached eGFR end points in a shorter time than those with a UACR < 30 mg/g (40% eGFR: 73.3% in 2.85 years vs. 7.9% in 4.6 years) (Supplementary Table S10), which is consistent with the observed associations between baseline UACR and annual eGFR decline. Moreover, KFRT occurred much more frequently in participants with UACR > 300 mg/g than in those with UACR < 30 mg/g (61.3% vs. 2.7%) (Supplementary Table S10).

Overall, 679 (13.0%) participants died, with an almost 2-fold higher mortality rate in men (Supplementary Table S9). A small subset of 25 participants (0.5%) experienced kidney death due to forgoing of KFRT. All-cause mortality was highest in DKD (24.9%), followed by HKD (14.9%), and lowest in PGD (5.5%) (Table 2). Mortality of individuals in the "fast decline" quintile was more than twice as high compared with those in the "slow decline" quintile (19.6% vs. 8.1%) (Table 1).

Overall, 221 participants (4.3%) had a rapid eGFR decline (> 5 ml/min per 1.73 m^2^/yr) with a mean annual rate of −6.28 (Table 2). Baseline UACR values were higher in participants with a rapid decline (Supplementary Table S10). The most common disease etiologies in the rapid eGFR decline group were ADPKD (33, 17.4%), followed by DKD (52, 6.9%) and PGD (70, 7.3%) with only a few cases observed in the HKD and MISC groups (Table 2). Interestingly, whereas the median eGFR slope between G1 and G4/5 was similar (−1.44 and −1.68), the proportion of rapid decliners was more than 3 times as high in the G1 group compared with the G4/5 group (10.1% vs. 3.0%) (Supplementary Table S5).

## Discussion

Our analyses provide a robust quantitative assessment of the heterogeneity of CKD progression using individually modeled eGFR slopes in a large cohort with different disease causes and severity. One of our main findings is a relatively low mean annual eGFR decline of −1.43 ml/min per 1.73 m^2^, only approximately 0.6 ml/min above the recently reported decline in healthy individuals.^31^ This modestly higher rate of decline occurred despite the fact that almost 70% of the study cohort met the KDIGO heat map criteria for "high" or "very high" risk for CKD progression at baseline and the mean baseline eGFR was < 50 ml/min per 1.73 m^2^.^24^ At the same time, we found a wide range of eGFR slopes, with a 5.04 ml/min difference of average slopes between Q1 and Q5.

Among patient characteristics associated with different rates of progression, the etiology of kidney disease stood out. Individuals with ADPKD showed an eGFR decline more than twice that of the overall cohort.^32^ According to study inclusion criteria, participants with ADPKD were enrolled if they had a reduced eGFR, and thus had already reached a progressive phase of eGFR decline.^16^ The second fastest decline was in participants with DKD, followed closely by those with PGD, many of whom were in stages G1 and G2. In contrast, individuals with HKD had an average eGFR decline close to physiological values and below the cohort average. Our findings in ADPKD and PGN align with a recent UK rare renal disease registry report showing higher disease progression rates in these etiologies.^33^

In DKD,^34^ there has been a notable increase in clinical trials in recent years.^35^, ^36^, ^37^ These trials usually select for rapid progression, primarily using higher UACR as inclusion criteria, which affects eGFR trajectories.^21^ Our result for unselected participants with DKD (−1.84), not on SGLT2 inhibitors during the observation period, aligns with recent trials of selected DKD participants under SGLT2 inhibitor treatment (−1.9).^37^ However, it contrasts with a meta-analysis of 66 studies from a diverse spectrum of interventions and populations showing faster declines overall (−3.17) and in the diabetic subgroup (−3.37), reflecting trial enrichment for more rapid progressors.^6^

Beyond CKD etiology, there was a strong association between UACR and eGFR slopes, with a more than 4-fold difference between A1 and A3 categories. These findings align with increased risks for kidney end points,^38^, ^39^, ^40^ as observed in our study and in previous research. In contrast to the association with UACR categories, we found no consistent difference in eGFR slopes across eGFR categories. The finding does not support previous conclusions of accelerated CKD progression with declining GFR,^21^ suggesting instead linear trajectories toward KF. However, the limited proportion of study participants in stage G4 limits the certainty of this finding.

In the current study, 4.2% of participants fulfilled the KDIGO definition of rapid CKD progression (> 5 ml/min per 1.73 m^2^).^22^^,^^23^ Whereas this observation across a large heterogenous CKD cohort supports this definition, our study suggests that rapid progression should be interpreted in the context of disease etiology and possibly other factors. Across etiology categories, the cutoff identifying the 5% with the fastest progression varied between 5.97 (ADPKD) and 3.57 (HKD) ml/min per 1.73 m^2^.

Our observations indicate that younger participants, particularly males with higher baseline UACR and classified into MISC, PGD, or DKD, exhibit a faster decline of eGFR. This aligns with findings from previous observations in the Chronic Renal Insufficiency Cohort and Modification of Diet in Renal Disease cohorts,^41^^,^^42^ as well as reported risk factors associated with accelerated eGFR decline.^43^ In the GCKD study, younger participants had higher eGFR values than older participants, except for those with ADPKD.

Our analysis indicates that sex differences in kidney function decline were significant only in certain subgroups, mirroring findings from the Chronic Renal Insufficiency Cohort study.^44^ Nonetheless, there are mixed reports on this topic, with some data suggesting that females have a slower decline,^43^ whereas others reported no difference or even a faster decline in females.^45^^,^^46^

Regarding the mean annual decline, 2 other CKD cohort studies reported similar results (−1.4 in Chronic Renal Insufficiency Cohort^47^ and SRR-CKD^43^), whereas steeper slopes were noted in other studies (−2.1 in KNOW-CKD^48^; −1.78 in CKD REIN^49^; and −1.79 for women and −2.09 for men in a pooled analysis^50^), likely reflecting enrollment criteria that favor those with more rapid disease progression. The progression of kidney disease is complex and different definitions (such as doubling serum creatinine, achieving KFRT or certain eGFR levels) have led to differences in the interpretation of study results.^2^^,^^22^^,^ 51, 52, 53 Because of the large number of eGFR values in the GCKD study, the modelling of CKD progression in our analysis is robust. In addition, commonly used kidney end points are provided for comparability and demonstrate consistent results with the eGFR slopes.

The number of eGFR estimates is a limiting factor for precise slope calculation and detecting the heterogeneity between and within subgroups. In clinical settings, many participants have an insufficient number for reliable slope calculations across the spectrum of CKD severity.^13^ In the current study, we combined eGFR estimates based on per-protocol creatinine measurements in a central laboratory with those obtained during routine clinical care. This allowed us to base our calculations on a median of 9 values during a 6.5 year observation period. Although this frequency is much lower than in prospective clinical studies, it is probably close to clinical practice in many cases. The use of different data sources had no influence on our results. Moreover, we employed mixed-effects modeling,^54^ allowing simultaneous modeling of baseline eGFR values and their trajectories, while accounting for correlations between measurements.^55^ This approach provides more accurate estimates than traditional single-slope analysis models, particularly in studies with high subject heterogeneity.^6^^,^^56^

Our findings have potential implications for clinical management. First, by highlighting disease heterogeneity, they illustrate the importance of comprehensive assessment of CKD. Associations of eGFR slopes with etiology emphasize the need to determine “cause” of CKD, as already recommended in the “CGA” categorization by KDIGO.^23^ Second, our findings suggest that individual eGFR slopes may be valuable as an additional, time-averaged kidney marker. As confirmed in our study, fast eGFR decline is associated with higher risks of kidney-related and non–kidney-related outcomes such as cardiovascular and all-cause mortality.^54^ This underscores the potential of stratifying patients according to prognosis to adjust management, care planning, and therapy.^57^^,^^58^ Third, in many patients, repetitive creatinine values are already determined in different settings, albeit not used for slope calculations. Widespread implementation of electronic health records capturing and integrating these data, as exemplified in our study, should facilitate slope estimations. Recently, eGFR slope analyses based on health checkup data were used in Japan to identify patients at risk for rapid CKD progresion.^59^ For patients fulfilling the inclusion criteria of our cohort, the slope estimates according to etiology, UACR, and eGFR stages may help to put individual data into perspective.

Among the strengths of this study is the large size of an ongoing cohort study of patients referred to nephrologists, offering comprehensive, high-density data on the progression of CKD over time. The wide range of kidney disease etiologies and the availability of subgroups of substantial size facilitated subgroup analyses. Standardized procedures include biomaterial handling and event adjudication, carried out by trained experts. The modeling approach allowed for estimating population-level effects while capturing variability between individual participants. Limitations of the study include eGFR range restrictions (inclusion criteria), Caucasian ethnicity only, and mandatory nephrological care, which confine generalizability. Potential biases include misclassification or possible cross-over between disease groups. Merging of all available creatinine values measured in the study under controlled conditions at follow-up visits (stable Roche autoanalyzer, enzymatic assay) and values collected from medical reports (enzymatic and Jaffe assay) increased the variability of the data.^60^ Intensified eGFR density due to adverse events (such as hospitalizations) could have influenced modeled eGFR trends. In contrast, the increased variability might lead to better transferability of results to data obtained in day-to-day clinical practice. The MISC group was large and heterogeneous with a high proportion of unknown etiology. Because the serum creatinine–based eGFR calculation, is influenced by factors other than kidney function, such as muscle mass, diet, and age. eGFR equations tend to overestimate the eGFR slope in younger patients and underestimate it in older patients.^61^ This variability can lead to misclassification of eGFR categories and the standardization of GFR to a body surface area can be problematic in individuals with extreme body sizes.^62^ Finally, given that some novel medications with the potential to slow CKD progression were not yet available during the observation period, eGFR slopes may be lower with the use of these agents in some subgroups.

In conclusion, eGFR slopes are a complementary instrument for quantitative assessment of CKD progression, reflecting disease heterogeneity and prognosis, and allowing refined stratification. Their potential to improve individualized management deserves further study.

## Appendix

### List of GCKD Study Investigators

University of Erlangen: Kai-Uwe Eckardt, Heike Meiselbach, Markus P. Schneider, Mario Schiffer, Hans-Ulrich Prokosch, Barbara Bärthlein, Andreas Beck, André Reis, Arif B. Ekici, Susanne Becker, Ulrike Alberth-Schmidt, Anke Weigel, Sabine Marschall, and Eugenia Schefler; University of Freiburg: Gerd Walz, Anna Köttgen, Ulla T. Schultheiß, Fruzsina Kotsis, Simone Meder, Erna Mitsch, and Ursula Reinhard; RWTH Aachen University: Jürgen Floege, Turgay Saritas, and Alice Groß; Charité, University Medicine Berlin: Elke Schaeffner, Seema Baid-Agrawal, and Kerstin Theisen; Hannover Medical School: Kai Schmidt-Ott; University of Heidelberg: Martin Zeier, Claudia Sommerer, and Mehtap Aykac; University of Jena: Gunter Wolf, Martin Busch, and Andy Steiner; Ludwig-Maximilians University of München: Thomas Sitter; University of Würzburg: Christoph Wanner, Vera Krane, Antje Börner-Klein, and Britta Bauer; Medical University of Innsbruck, Division of Genetic Epidemiology: Florian Kronenberg, Julia Raschenberger, Barbara Kollerits, Lukas Forer, Sebastian Schönherr, and Hansi Weissensteiner; University of Regensburg, Institute of Functional Genomics: Peter Oefner and Wolfram Gronwald; Institute of Medical Biometry, Informatics and Epidemiology, Medical Faculty, University of Bonn: Matthias Schmid and Jennifer Nadal.

## Disclosure

ES received consulting fees from AstraZeneca, received honoraria as a speaker from the National Kidney Foundation, and is a board member of German Society of Nephrology and a KDIGO Working group. KUE received grant funding from BAYER, Evotec, Travere, and AstraZeneca; received consulting fees from Akebia, AstraZeneca, BAYER, Boehringer Ingelheim, Medice, and Novartis; and participated on a Data Safety Monitoring Board or Advisory Board for AstraZeneca. All the other authors declared no competing interests.

## Supplementary Material

Supplementary File (PDF) **Supplementary References. Material S1.** Data collection and measurements. **Material S2.** Model equations. **Figure S1.** Flowchart of GCKD study participants (*n*) and observed eGFR values (v) used for analyses. **Figure S2.** An example of an eGFR slope with the end points of interest for eGFR decline over 6.5 years of follow-up. **Figure S3.** Observed individual and mean eGFR slopes by disease etiology, age and sex over 6.5 years follow-up. **Table S1.** Characteristics of participants that are not included in Model 2 due to missing UACR information at baseline. **Table S2** Baseline characteristics of GCKD participants stratified by disease etiology. **Table S3.** Baseline characteristics of GCKD study participants stratified by baseline eGFR categories. **Table S4** Baseline characteristics of GCKD participants stratified by baseline UACR categories. **Table S5.** Model-based eGFR slopes, GFR related and other end points stratified by baseline eGFR**. Table S6**. Model characteristics. **Table S7.** Sensitivity analysis: model characteristics using only data available at follow-up visits. **Table S8.** Model characteristics of interaction models. **Table S9.** Number of participants reaching kidney related end points during 6.5 years of follow-up. **Table S10.** Model-based eGFR slopes, GFR-related and other end points stratified by baseline UACR categories. STROBE statement.

## Footnotes

Supplementary File (PDF)
Supplementary References. **Material S1.** Data collection and measurements. **Material S2**. Model equations. **Figure S1.** Flowchart of GCKD study participants (*n*) and observed eGFR values (v) used for analyses. **Figure S2.** An example of an eGFR slope with the end points of interest for eGFR decline over 6.5 years of follow-up. **Figure S3.** Observed individual and mean eGFR slopes by disease etiology, age and sex over 6.5 years follow-up. **Table S1.** Characteristics of participants that are not included in Model 2 due to missing UACR information at baseline. **Table S2.** Baseline characteristics of GCKD participants stratified by disease etiology. **Table S3.** Baseline characteristics of GCKD study participants stratified by baseline eGFR categories. **Table S4.** Baseline characteristics of GCKD participants stratified by baseline UACR categories. **Table S5.** Model-based eGFR slopes, GFR related and other end points stratified by baseline eGFR. **Table S6.** Model characteristics. **Table S7.** Sensitivity analysis: model characteristics using only data available at follow-up visits. **Table S8.** Model characteristics of interaction models. **Table S9.** Number of participants reaching kidney related end points during 6.5 years of follow-up. **Table S10.** Model-based eGFR slopes, GFR-related and other end points stratified by baseline UACR categories. STROBE statement.
