# Supplementary material for: Heterogeneity of Estimated GFR Slopes According to Etiology, Estimated GFR and Urinary Albumin-to-Creatinine Ratio in a Large Cohort of Patients With CKD
Source: Kidney Int Rep. 2025 Nov 20;11(2):103696. doi: 10.1016/j.ekir.2025.11.021 (PMC12800589; doi:10.1016/j.ekir.2025.11.021)
Supplement: Supplementary File (PDF) — Supplementary References. Material S1. Data collection and measurements. Material S2. Model equations. Figure S1. Flowchart of GCKD study participants (n) and observed eGFR values (v) used for analyses. Figure S2. An example of an eGFR slope with the end points of interest for eGFR decline over 6.5 years of follow-up. Figure S3. Observed individual and mean eGFR slopes by disease etiology, age and sex over 6.5 years follow-up. Table S1. Characteristics of participants that are not included in Model 2 due to missing UACR information at baseline. Table S2 Baseline characteristics of GCKD participants stratified by disease etiology. Table S3. Baseline characteristics of GCKD study participants stratified by baseline eGFR categories. Table S4 Baseline characteristics of GCKD participants stratified by baseline UACR categories. Table S5. Model-based eGFR slopes, GFR related and other end points stratified by baseline eGFR. Table S6. Model characteristics. Table S7. Sensitivity analysis: model characteristics using only data available at follow-up visits. Table S8. Model characteristics of interaction models. Table S9. Number of participants reaching kidney related end points during 6.5 years of follow-up. Table S10. Model-based eGFR slopes, GFR-related and other end points stratified by baseline UACR categories. STROBE statement. [file mmc1.pdf]

Supplementary material

Heterogeneity of eGFR slopes according to etiology, eGFR and UACR in a large cohort of CKD patients

Charlotte Behning*, Ulla T. Schultheiss*, Jennifer Nadal, Heike Meiselbach, Sebastian Schönherr, Lukas Forer, Elke Schäffner, Vera Krane, Markus P. Schneider, Matthias Schmid, Florian Kronenberg, Anna Köttgen, Kai-Uwe Eckardt, Fruzsina Kotsis, on behalf of the GCKD study investigators

*these authors contributed equally to this project

Content

Content

Material S1. Data collection and measurements....3

Material S2. Model equations 4-5

Figure S1. Flowchart of GCKD study participants (n) and observed eGFR values (v) used for analyses....6

Figure S2. An example of an eGFR slope with the endpoints of interest for eGFR decline over 6.5 years of follow-up. 7

Figure S3. Observed individual and mean eGFR slopes by disease etiology, age and sex over 6.5 years follow-up. 8

Table S1. Characteristics of participants that are not included in Model 2 due to missing UACR information at baseline. 9

Table S2 Baseline characteristics of GCKD participants stratified by disease etiology. 10

Table S3. Baseline characteristics of GCKD study participants stratified by baseline eGFR categories. 12

Table S4 Baseline characteristics of GCKD participants stratified by baseline UACR categories. 14

Table S5. Model-based eGFR slopes, GFR related and other endpoints stratified by baseline eGFR 16

Table S6. Model characteristics....17

Table S7. Sensitivity analysis: Model characteristics using only data available at follow-up visits....18

Table S8. Model characteristics of interaction models. 19

Table S9. Number of participants reaching kidney related endpoints during 6.5 years of follow-up....20

Table S10. Model-based eGFR slopes, GFR related and other endpoints stratified by baseline UACR categories. 21

Note S1. Current GCKD Investigators and Collaborators with the GCKD Study are: 22

Supplementary References. 23

STROBE Statement 24

Material S1. Data collection and measurements

Participants underwent annual follow-up visits, alternating between in-person and telephone visits. At baseline and at each in-person follow-up visit, serum and urine samples were collected, frozen, and transported in a standardized fashion to a central biobank where they were stored at -80°C for future analyses.[S1] To measure serum creatinine an IDMS traceable enzymatic assay (Creatinine Plus, Roche) was used. Urine creatinine for the calculation of UACR (mg albumin /g creatinine) was measured with the same assay as in serum, and urine albumin was measured with the ALBU-XS assay.[24] All discharge reports of hospitalizations were collected after the follow-up visits. In addition, available serum creatinine values (measured by the treating nephrologist or in hospitals) were also collected, with the source of data (outpatient, in hospital) and the measurement unit (mg/dL, mmol/L) specified. From these creatinine values (medical report values) and from the measured creatinine values (study visit values), eGFR (mL/min/1.73 m^2) was calculated using the Chronic Kidney Disease Epidemiology Collaboration (CKD-EPI 2009) formula[S2] as implemented in the R package *nephro.*[S3] According to the KDIGO classification participants were categorized by baseline eGFR (G1-G5) and UACR (A1-A3).[25] The presumed leading cause of CKD (disease etiology) was provided for each participant by the treating nephrologist at baseline.

Material S2. Model equations

All models were fitted in R using the lme4 package. In all models, a chronic linear eGFR decline was assumed.

Model 1

Without baseline covariates, using a particpant-specific random intercept and slope term.

$$y_{it} = \beta_0 + \beta_{time} \cdot t + \alpha_{0,i} + \alpha_{time,i} \cdot t + \varepsilon_{i,t} \ ,$$

with

y_{it}: eGFR value of participant $i = 1, \ldots, 5214$ at follow-up time $t \in [0, 6.5]$ in years.

β_0: Fixed intercept term/ population level intercept.

β_{time}: Fixed effect for follow-up time/ population level slope.

$\alpha_{0,i}$: Random intercept term/participant level intercept, $\alpha_0 \sim \mathcal{N}(0, \sigma_0^2)$.

$\alpha_{time,i}$: Random effect for slope/participant level slope $\alpha_{time} \sim \mathcal{N}(0, \sigma_{time}^2)$.

$\varepsilon_{i,t}$: Residuals with $\varepsilon \sim \mathcal{N}(0, \sigma_\varepsilon^2)$.

Model 2

With baseline covariates sex, age, UACR and disease etiology, using a particpant-specific random intercept and slope term.

$$\begin{aligned}
y_{it} = &\beta_0 + \beta_{sex} \cdot x_{sex} + \beta_{age} \cdot x_{age} + \beta_{UACR_1} \cdot I(30 \leq x_{UACR} < 300) \\
&+ \beta_{UACR_2} \cdot I(300 \leq x_{UACR} \leq 3000) + \beta_{UACR_3} \cdot I(x_{UACR} > 3000) \\
&+ \beta_{PGD} \cdot I(x_{etiology} = PGD) + \beta_{DKD} \cdot I(x_{etiology} = DKD) \\
&+ \beta_{ADPKD} \cdot I(x_{etiology} = ADPKD) + \beta_{other} \cdot I(x_{etiology} = other) \\
&+ \big(\beta_{time} + \beta_{time \times sex} \cdot x_{sex} + \beta_{time \times age} \cdot x_{age} + \beta_{time \times UACR_1} \cdot I(30 \leq x_{UACR} < 300) \\
&+ \beta_{time \times UACR_2} \cdot I(300 \leq x_{UACR} \leq 3000) + \beta_{time \times UACR_3} \cdot I(x_{UACR} > 3000) \\
&+ \beta_{time \times PGD} \cdot I(x_{etiology} = PGD) + \beta_{time \times DKD} \cdot I(x_{etiology} = DKD) \\
&+ \beta_{time \times ADPKD} \cdot I(x_{etiology} = ADPKD)\big) \cdot t \\
&+ \alpha_{0,i} + \alpha_{time,i} \cdot t \\
&+ \varepsilon_{i,t} \ ,
\end{aligned}$$

with

y_{it}: eGFR value of participant $i = 1, \ldots, 5122$ at follow-up time $t \in [0, 6.5]$ in years.

β_0: Fixed intercept term. Please note, in contrast to Model 1, the intercept has to be interpreted w.r.t the reference categories (sex=male, age=0, UACR $<$ 30, etiology= HKD).

$\beta_{time}, \alpha_{0,i}, \alpha_{time,i}$: see above.

$\beta_{sex}, \beta_{age}, \beta_{UACR_1}, \beta_{UACR_2}, \beta_{UACR_3}, \beta_{PGD}, \beta_{DKD}, \beta_{ADPKD}, \beta_{other}$: Fixed covariate effects/population level effects of covariates affecting the intercept.

$I()$: Indicator function.

$x_{sex}, x_{age}, x_{UACR}, x_{etiology}$: Baseline covariates. Age is given in years. The reference categories (sex=male, age=0, etiologyHKD).

$\beta_{time \times sex}, \beta_{time \times age}, \beta_{time \times UACR_1}, \beta_{time \times UACR_2}, \beta_{time \times UACR_3}, \beta_{time \times PGD}, \beta_{time \times DKD}, \beta_{time \times ADPKD}, \beta_{other}$: Fixed effect for the interaction of covariates with the follow-up time/ covariate-effects affecting the slope.

Models 3A - 3E

With baseline covariates sex, age groups (older vs younger than the median age), and sex and age interaction. Separate models are fitted for each disease etiology.

$$\begin{aligned} y_{it} = & \beta_0 + \beta_{sex} \cdot x_{sex} + \beta_{age} \cdot I(x_{age} \geq 63) + \beta_{sex \times age} \cdot x_{sex} \cdot I(x_{age} \geq 63) \\ & + \big(\beta_{time} + \beta_{time \times sex} \cdot x_{sex} + \beta_{time \times age} \cdot I(x_{age} \geq 63) \\ & + \beta_{time \times sex \times age} \cdot x_{sex} \cdot I(x_{age} \geq 63)\big) \cdot t + \\ & + \alpha_{0,i} + \alpha_{time,i} \cdot t \\ & + \varepsilon_{i,t} \ , \end{aligned}$$

with

y_{it}: eGFR value of participant $i = 1, \ldots, n_{etiology}$ of disease etiology group at follow-up time $t \in [0, 6.5]$ in years.

$\beta_0, \beta_{sex}, \beta_{time}$: Fixed effects as defined above

β_{age}: Fixed effect for age group older 63.

$\beta_{sex \times age}$: Fixed effect for the interaction of sex and age older 63 years.

$\beta_{time \times sex}, \beta_{time \times age}$: Fixed effect for the interaction of covariates with the follow-up time/ covariate-effects affecting the slope.

$\beta_{time \times sex \times age}$: Fixed effect for the interaction of sex and age with the follow-up time / covarites-interaction affecting the slope on population level.

$\alpha_{0,i}, \alpha_{time,i}, \varepsilon$: see above.

Figure S1. Flowchart of GCKD study participants (n) and observed eGFR values (v) used for analyses.

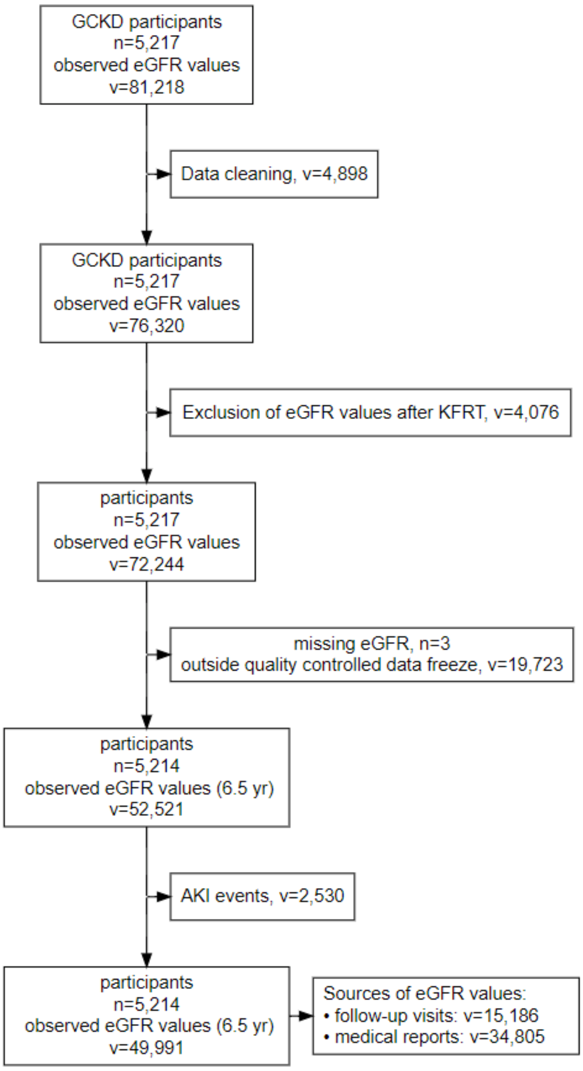

Footnote/abbreviations: GCKD=German Chronic Kidney Disease study, eGFR=estimated glomerular filtration rate, v=number of observed eGFR values, n=number of participants, yr=years, KFRT: kidney failure treated by kidney replacement therapy, AKI: acute kidney injury. A total of n=5,217 patients with v=81,218 eGFR values were available in the data freeze at 03/2022. After data cleaning, e.g. removal of duplicated entries, v=76,320 eGFR values remained in the dataset. Further, 4,076 eGFR values that were obtained after reaching KFRT, were excluded, leaving v=72,244 eGFR values. After these steps, no usable data were available for 3 participants and these were removed from the analysis data set. To ensure a consistent quality controlled dataset, all values obtained after 6.5 years of follow-up were further removed, leaving v=52,521 eGFR values for n=5,214 participants. Further 2,530 eGFR values that were obtained during AKI events (±7 days) were removed. The remaining v=49,991 were collected during follow-up visits (v=15,186) or from medical report (v=34,805).

Figure S2. An example of an eGFR slope with the endpoints of interest for eGFR decline over 6.5 years of follow-up.

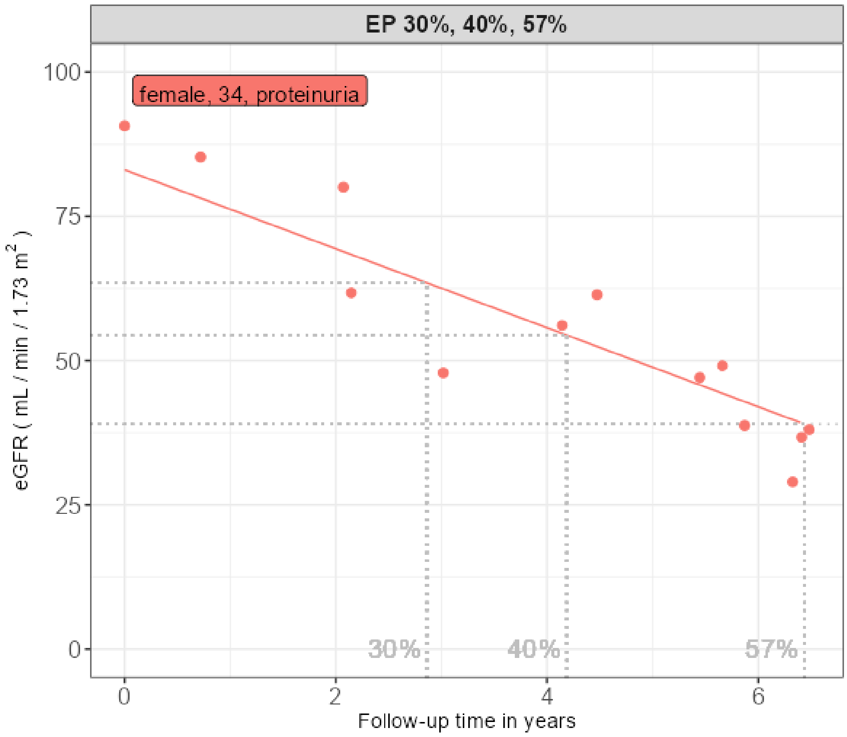

Footnote/abbreviations: The red points indicate observed eGFR (=estimated glomerular filtration) values, while the solid red line represents the modelled eGFR slope of an individual diagnosed with PGD (=primary glomerular disease), and the decline in eGFR for this participant. The reference lines (shown in grey) highlight the endpoints (EP) 30%, 40% and 57% eGFR decline compared to the participant's baseline eGFR. The modelled eGFR is obtained from a linear mixed-effects model, which simultaneously models all participants.

Figure S3. Observed individual and mean eGFR slopes by disease etiology, age and sex over 6.5 years follow-up.

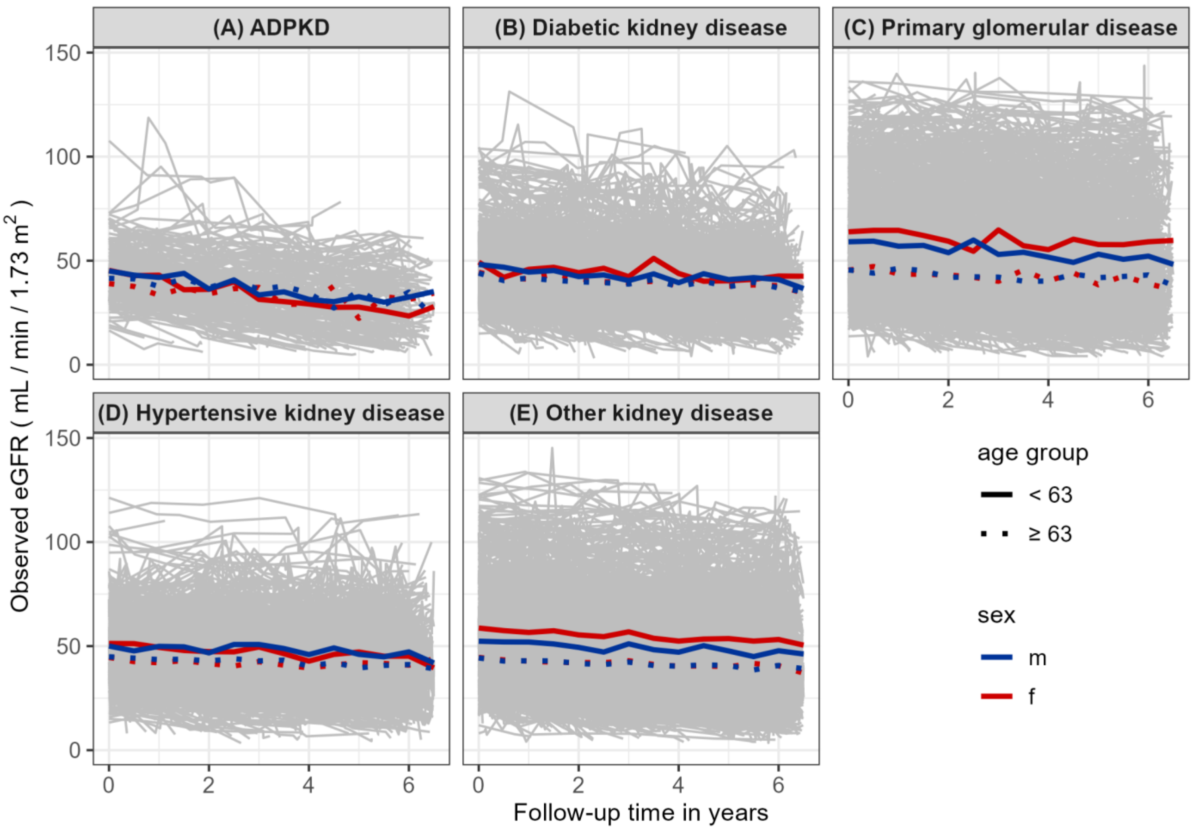

Number participants	ADPKD		Diabetic kidney disease		Primary glomerular disease		Hypertensive kidney disease		Other kidney disease	
**Age**	m	f	m	f	m	f	m	f	m	f
**< 63 years**	72	72	162	84	439	253	239	128	484	507
**≥ 63 years**	29	18	374	163	194	91	540	291	598	476

Footnote/abbreviations: Participants are stratified by disease etiology. Using all available eGFR values over 6.5 years of follow-up, the observed eGFR values are averaged for each participant in 6-month time windows and further averaged across disease etiology, sex and age group. Age subgroups are separated at the median age of 63 years. m: male, f: female. The table below shows the number of participants within each subgroup defined by disease etiology, sex and age group. ADPKD: autosomal dominant polycystic kidney disease. The total participant number is based on the cleaned dataset.

Table S1. Characteristics of participants that are not included in Model 2 due to missing UACR information at baseline.

	**NA - not modelled** (N=92)	**Overall** (N=5,214)
**Age (years)**		
Mean (SD)	60.7 (11.7)	60.1 (12.0)
**Sex**		
male	52 (56.5%)	3131 (60.0%)
**eGFR categories** (mL/min/1.73m^2)		
G4+G5: <30	8 (8.7%)	504 (9.7%)
G3b: 30-44	28 (30.4%)	1905 (36.5%)
G3a: 45-59	36 (39.1%)	1713 (32.9%)
G2: 60-89	16 (17.4%)	861 (16.5%)
G1: >=90	4 (4.3%)	231 (4.4%)
**Inclusion criteria**		
eGFR	87 (94.6%)	4774 (91.6%)
albuminuria	5 (5.4%)	440 (8.4%)
**Medication**		
ACEI	41 (44.6%)	2471 (47.4%)
ARBs	40 (43.5%)	2351 (45.1%)
Missing	1 (1.1%)	35 (0.7%)
**Disease etiology**		
ADPKD	1 (1.1%)	191 (3.7%)
Diabetic kidney disease	24 (26.1%)	783 (15.0%)
Primary glomerular disease	12 (13.0%)	977 (18.7%)
Hypertensive kidney disease	20 (21.7%)	1198 (23.0%)
MISC	35 (38.0%)	2065 (39.6%)
**Kidney death**	1 (1.1%)	25 (0.5%)
**KFRT**	6 (6.5%)	487 (9.3%)
**All-cause death**	20 (21.7%)	679 (13.0%)

Footnote/abbreviations: eGFR (estimated glomerular filtration rate in mL/min/1.73 m^2) categories (at baseline): G1-G5 expressed in mL/min/1.73 m2. UACR categories (at baseline): urinary albumin/creatinine. Inclusion criteria: eGFR 30–60 mL/min/1.73m^2 or albuminuria, if eGFR >60 mL/min/1.73 m^2 and UACR (urinary albumin to creatinine ratio) >300 mg/g or >300 mg/day. Kidney death: death due to forgoing of dialysis. kidney failure: kidney failure with replacement therapy or kidney death. ADPKD: autosomal dominant polycystic kidney disease. MISC: other underlying kidney disease. Baseline characteristics are reported as mean with standard deviation (±SD), median with 25% and 75% quartiles (IQR: Q25, Q75) or as absolute and relative frequencies. KFRT: KF treated by kidney replacement therapy. ACEI: Angiotensin-converting-enzyme inhibitors, ARBs: Angiotensin receptor blockers.

Table S2 Baseline characteristics of GCKD participants stratified by disease etiology.

	**ADPKD** (N=191)	**Diabetic kidney disease** (N=783)	**Primary glomerular disease** (N=977)	**Hypertensive kidney disease** (N=1198)	**MISC** (N=2065)	**Overall** (N=5214)
**Age** (years)						
Mean (SD)	54.1 (10.7)	64.5 (8.02)	52.7 (13.6)	64.7 (8.5)	59.8 (12.1)	60.1 (12.0)
**Sex**						
male	101 (52.9%)	536 (68.5%)	633 (64.8%)	779 (65.0%)	1082 (52.4%)	3131 (60.0%)
**eGFR categories** (mL/min/1.73 m^2)						
G4+G5: <30	22 (11.5%)	95 (12.1%)	100 (10.2%)	101 (8.4%)	186 (9.0%)	504 (9.7%)
G3b: 30-44	88 (46.1%)	325 (41.5%)	285 (29.2%)	480 (40.1%)	727 (35.2%)	1905 (36.5%)
G3a: 45-59	60 (31.4%)	257 (32.8%)	246 (25.2%)	442 (36.9%)	708 (34.3%)	1713 (32.9%)
G2: 60-89	20 (10.5%)	89 (11.4%)	219 (22.4%)	166 (13.9%)	367 (17.8%)	861 (16.5%)
G1: >=90	1 (0.5%)	17 (2.2%)	127 (13.0%)	9 (0.8%)	77 (3.7%)	231 (4.4%)
**UACR** (mg/g)						
A1 <30	72 (37.7%)	284 (36.3%)	133 (13.6%)	702 (58.6%)	997 (48.3%)	2188 (42.0%)
A2 [30,299]	94 (49.2%)	237 (30.3%)	282 (28.9%)	308 (25.7%)	570 (27.6%)	1491 (28.6%)
A3 [300,3000]	24 (12.6%)	211 (26.9%)	475 (48.6%)	161 (13.4%)	426 (20.6%)	1297 (24.9%)
>3000	0 (0%)	27 (3.4%)	75 (7.7%)	7 (0.6%)	37 (1.8%)	146 (2.8%)
Missing	1 (0.5%)	24 (3.1%)	12 (1.2%)	20 (1.7%)	35 (1.7%)	92 (1.8%)
**Inclusion criteria**						
G3, A1-A3	187 (97.9%)	746 (95.3%)	744 (76.2%)	1174 (98.0%)	1923 (93.1%)	4774 (91.6%)
G1-2, A3	4 (2.1%)	37 (4.7%)	233 (23.8%)	24 (2.0%)	142 (6.9%)	440 (8.4%)
**Medication**						
ACEi	81 (42.4%)	405 (51.7%)	566 (57.9%)	544 (45.4%)	875 (42.4%)	2471 (47.4%)
ARBs	106 (55.5%)	370 (47.3%)	511 (52.3%)	548 (45.7%)	816 (39.5%)	2351 (45.1%)
Missing	1 (0.5%)	0 (0%)	6 (0.6%)	2 (0.2%)	26 (1.3%)	35 (0.7%)
**Kidney biopsy**						
yes	6 (3.1%)	33 (4.2%)	789 (80.8%)	92 (7.7%)	447 (21.6%)	1367 (26.2%)
no	185 (96.9%)	750 (95.8%)	187 (19.1%)	1104 (92.2%)	1617 (78.3%)	3843 (73.7%)
Missing	0 (0%)	0 (0%)	1 (0.1%)	2 (0.2%)	1 (0.0%)	4 (0.1%)
**Number of eGFR values per participant in 6.5 years**						
Median [Q25, Q75]	9.00 [6.50, 11.0]	9.00 [6.00, 12.0]	9.00 [7.00, 12.0]	10.0 [7.00, 12.0]	10.0 [7.00, 12.0]	9.00 [7.00, 12.0]
Follow-up visits	3.00 [2.00, 4.00]	3.00 [2.00, 4.00]	3.00 [2.00, 4.00]	3.00 [2.00, 4.00]	3.00 [2.00, 4.00]	3.00 [2.00, 4.00]

	ADPKD (N=191)	Diabetic kidney disease (N=783)	Primary glomerular disease (N=977)	Hypertensive kidney disease (N=1198)	MISC (N=2065)	Overall (N=5214)
Medical reports	6.00 [4.00, 7.00]	6.00 [4.00, 9.00]	6.00 [4.00, 8.00]	6.00 [4.00, 8.00]	6.00 [4.00, 9.00]	6.00 [4.00, 8.00]
**Number of eGFR values per participant per year**						
Median [Q25, Q75]	1.82 [1.50, 2.28]	2.00 [1.56, 2.68]	1.71 [1.42, 2.16]	1.81 [1.50, 2.40]	1.77 [1.47, 2.39]	1.8 [1.49, 2.37]

Footnote/abbreviations: Participants are stratified by disease etiology. eGFR (=estimated glomerular filtration rate) categories at baseline G1-G5 (expressed in mL/min/1.73 m^2). UACR (at baseline): urinary albumin/creatinine. Number of eGFR values including values from follow-up visits and medical reports (see **Figure S1**). ADPKD: autosomal dominant polycystic kidney disease. MISC: other kidney disease. N: number of participants with available values. Mean values are reported with standard deviation (±SD), median with 25% and 75% quartiles (interquartile range IQR: Q25, Q75) or as absolute and relative frequencies. The overall participant number is based on the complete case cohort set. ACEI: Angiotensin-converting-enzyme inhibitors, ARBs: Angiotensin receptor blockers.

Table S3. Baseline characteristics of GCKD study participants stratified by baseline eGFR categories.

	**Overall** (N=5214)	**G4+G5: <30** (N=504)	**G3b: 30-44** (N=1905)	**G3a: 45-59** (N=1713)	**G2: 60-89** (N=861)	**G1: >=90** (N=231)
**Age** (years)						
Mean (SD)	60.1 (12.0)	63.1 (10.6)	62.7 (10.4)	61.2 (10.4)	55.3 (12.8)	41.6 (12.6)
**Sex**						
male	3131 (60.0%)	312 (61.9%)	1170 (61.4%)	1064 (62.1%)	467 (54.2%)	118 (51.1%)
**Disease etiology**						
ADPKD	191 (3.7%)	22 (4.4%)	88 (4.6%)	60 (3.5%)	20 (2.3%)	1 (0.4%)
Diabetic kidney disease	783 (15.0%)	95 (18.8%)	325 (17.1%)	257 (15.0%)	89 (10.3%)	17 (7.4%)
Primary glomerular disease	977 (18.7%)	100 (19.8%)	285 (15.0%)	246 (14.4%)	219 (25.4%)	127 (55.0%)
Hypertensive kidney disease	1198 (23.0%)	101 (20.0%)	480 (25.2%)	442 (25.8%)	166 (19.3%)	9 (3.9%)
MISC	2065 (39.6%)	186 (36.9%)	727 (38.2%)	708 (41.3%)	367 (42.6%)	77 (33.3%)
**UACR** (mg/g)						
A1 <30	2188 (42.0%)	142 (28.2%)	770 (40.4%)	873 (51.0%)	378 (43.9%)	25 (10.8%)
A2 [30,299]	1491 (28.6%)	163 (32.3%)	602 (31.6%)	461 (26.9%)	216 (25.1%)	49 (21.2%)
A3 [300,3000]	1297 (24.9%)	162 (32.1%)	456 (23.9%)	314 (18.3%)	231 (26.8%)	134 (58.0%)
>3000	146 (2.8%)	29 (5.8%)	49 (2.6%)	29 (1.7%)	20 (2.3%)	19 (8.2%)
Missing	92 (1.8%)	8 (1.6%)	28 (1.5%)	36 (2.1%)	16 (1.9%)	4 (1.7%)
**Inclusion criteria**						
G3, A1-A3	4774 (91.6%)	501 (99.4%)	1895 (99.5%)	1674 (97.7%)	676 (78.5%)	28 (12.1%)
G1-2, A3	440 (8.4%)	3 (0.6%)	10 (0.5%)	39 (2.3%)	185 (21.5%)	203 (87.9%)
**Medication**						
ACEI	2471 (47.4%)	243 (48.2%)	899 (47.2%)	822 (48.0%)	369 (42.9%)	138 (59.7%)
ARBs	2351 (45.1%)	254 (50.4%)	900 (47.2%)	741 (43.3%)	362 (42.0%)	94 (40.7%)
Missing	35 (0.7%)	2 (0.4%)	2 (0.1%)	12 (0.7%)	15 (1.7%)	4 (1.7%)
**Number of eGFR values per participant in 6.5 years**						
Median [Q25, Q75]	9.00 [7.00, 12.0]	10.0 [5.00, 13.0]	10.0 [7.00, 13.0]	10.0 [7.00, 12.0]	9.00 [7.00, 11.0]	9.00 [7.00, 11.0]
Follow-up visits	3.00 [2.00, 4.00]	2.00 [1.00, 4.00]	3.00 [2.00, 4.00]	3.00 [2.00, 4.00]	3.00 [2.00, 4.00]	3.00 [2.00, 4.00]
Medical reports	6.00 [4.00, 8.00	6.00 [6.00, 9.25]	6.00 [4.00, 9.00]	6.00 [4.00, 8.00]	6.00 [4.00, 8.00]	6.00 [4.00, 8.00]]

Footnote/abbreviations: Participants were stratified by baseline eGFR categories (estimated glomerular filtration rate in mL/min/1.73 m^2) according to KDIGO stages: G1-G5 and UACR (urinary albumin to creatinine ratio). Number of eGFR values including values from follow-up visits and medical reports (see **Figure S1**). ADPKD: autosomal dominant polycystic kidney disease. MISC: other underlying kidney disease. Baseline characteristics are reported as mean with standard deviation (±SD), median with 25% and 75% quartiles (interquartile range IQR: Q25, Q75) or

as absolute and relative frequencies. ACEI: Angiotensin-converting-enzyme inhibitors, ARBs: Angiotensin receptor blockers.

Table S4 Baseline characteristics of GCKD participants stratified by baseline UACR categories.

	**<30 mg/g** (N=2188)	**[30,299] mg/g** (N=1491)	**[300,3000] mg/g** (N=1297)	**>3000 mg/g** (N=146)	**Overall** (N=5122)
**Age** (years)					
Mean (SD)	63.3 (9.5)	59.9 (11.9)	55.5 (13.5)	52.8 (14.5)	60.1 (12.0)
Sex					
male	1150 (52.6%)	952 (63.8%)	882 (68.0%)	95 (65.1%)	3079 (60.1%)
**eGFR categories** (mL/min/1.73 m^2)					
G4+G5: <30	142 (6.5%)	163 (10.9%)	162 (12.5%)	29 (19.9%)	496 (9.7%)
G3b: 30-44	770 (35.2%)	602 (40.4%)	456 (35.2%)	49 (33.6%)	1877 (36.6%)
G3a: 45-59	873 (39.9%)	461 (30.9%)	314 (24.2%)	29 (19.9%)	1677 (32.7%)
G2: 60-89	378 (17.3%)	216 (14.5%)	231 (17.8%)	20 (13.7%)	845 (16.5%)
G1: >=90	25 (1.1%)	49 (3.3%)	134 (10.3%)	19 (13.0%)	227 (4.4%)
**Disease etiology**					
ADPKD	72 (3.3%)	94 (6.3%)	24 (1.9%)	0 (0%)	190 (3.7%)
Diabetic kidney disease	284 (13.0%)	237 (15.9%)	211 (16.3%)	27 (18.5%)	759 (14.8%)
Primary glomerular disease	133 (6.1%)	282 (18.9%)	475 (36.6%)	75 (51.4%)	965 (18.8%)
Hypertensive kidney disease	702 (32.1%)	308 (20.7%)	161 (12.4%)	7 (4.8%)	1178 (23.0%)
MISC	997 (45.6%)	570 (38.2%)	426 (32.8%)	37 (25.3%)	2030 (39.6%)
**Inclusion criteria**					
G3, A1-A3	2167 (99.0%)	1393 (93.4%)	1017 (78.4%)	110 (75.3%)	4687 (91.5%)
G1-2, A3	21 (1.0%)	98 (6.6%)	280 (21.6%)	36 (24.7%)	435 (8.5%)
**Medication**					
ACEI	914 (41.8%)	723 (48.5%)	704 (54.3%)	89 (61.0%)	2430 (47.4%)
ARBs	915 (41.8%)	660 (44.3%)	659 (50.8%)	77 (52.7%)	2311 (45.1%)
Missing	20 (0.9%)	7 (0.5%)	4 (0.3%)	3 (2.1%)	34 (0.7%)
**Number of eGFR values per participant in 6.5 years**					
Median [Q25, Q75]	10.0 [7.0, 12.0]	10.0 [7.0, 12.0]	9.0 [7.0, 12.0]	8.0 [4.25, 12.0]	10.0 [7.0, 12.0]
Follow-up visits	3.00 [2.00, 4.00]	3.00 [2.00, 4.00]	3.00 [2.00, 4.00]	2.00 [1.00, 3.00]	3.00 [2.00, 4.00]
Medical reports	6.00 [4.00, 8.00]	6.00 [4.00, 9.00]	6.00 [4.00, 8.00]	6.00 [3.00, 8.00]	6.00 [4.00, 8.00]

Footnote/abbreviations: Participants are stratified by UACR categories (at baseline): G1-G5 expressed in mL/min/1.73 m^2. UACR categories (at baseline): urinary albumin/creatinine. Number of eGFR values including values from follow-up visits and medical reports (see **Figure S1**). ADPKD: autosomal dominant polycystic kidney disease. MISC: other kidney disease. N: number of participants with available values. Mean reported with standard

deviation (±SD), median with 25% and 75% quartiles (interquartile range IQR: Q25, Q75) or as absolute and relative frequencies. The total participant number is based on the complete case cohort dataset. ACEI: Angiotensin-converting-enzyme inhibitors, ARBs: Angiotensin receptor blockers.

Table S5. Model-based eGFR slopes, GFR related and other endpoints stratified by baseline eGFR

		G4+G5: <30 (N=496)	G3b: 30-44 (N=1877)	G3a: 45-59 (N=1677)	G2: 60-89 (N=845)	G1: >=90 (N=227)	Overall (N=5122)
**Model (2) slope**	**eGFR slope rate**						
	Mean (SD)	-1.71 (1.73)	-1.44 (1.71)	-1.27 (1.81)	-1.43 (2.11)	-1.97 (2.51)	-1.43 (1.86)
	Median [Q25, Q75]	-1.68 [-2.90, -0.54]	-1.26 [-2.39, -0.41]	-1.01 [-2.16, -0.21]	-1.04 [-2.44, -0.16]	-1.44 [-2.62, -0.32]	-1.18 [-2.41, -0.31]
	Rapid decline	15 (3.0%)	60 (3.2%)	74 (4.4%)	49 (5.8%)	23 (10.1%)	221 (4.3%)
**Estimated EPs derived from Model (2)**	**Absolut and relative frequency reaching estimated EP:** n (%)						
	**30% eGFR**	252 (50.8%)	678 (36.1%)	433 (25.8%)	209 (24.7%)	41 (18.1%)	1613 (31.5%)
	4**0% eGFR**	206 (41.5%)	488 (26.0%)	257 (15.3%)	125 (14.8%)	25 (11.0%)	1101 (21.5%)
	**57% eGFR**	151 (30.4%)	274 (14.6%)	121 (7.2%)	46 (5.4%)	11 (4.8%)	603 (11.8%)
	Missing	2 (0.4%)	0 (0%)	0 (0%)	0 (0%)	0 (0%)	2 (0.0%)
	**Time EP reached**: Median [Q25, Q75]						
	**30% eGFR**	3.41 [2.04, 4.72]	3.64 [2.49, 5.02]	4.01 [2.69, 5.23]	3.87 [2.58, 4.97]	3.29 [2.11, 5.26]	3.73 [2.47, 5.03]
	**40% eGFR**]	3.84 [2.54, 4.86]	4.25 [3.06, 5.26]	4.33 [3.20, 5.37]	4.67 [3.12, 5.63]	4.19 [3.09, 5.21]	4.22 [3.02, 5.25]
	**57% eGFR**	4.37 [3.36, 5.61]	4.78 [3.89, 5.58]	5.08 [4.05, 5.75]	4.79 [3.51, 5.51]	4.90 [3.78, 5.71]	4.76 [3.66, 5.65]
**Observed EPs**	**Kidney death**	8 (1.6%)	9 (0.5%)	6 (0.4%)	1 (0.1%)	0 (0%)	24 (0.5%)
	**KFRT**	166 (33.5%)	232 (12.4%)	72 (4.3%)	22 (2.6%)	3 (1.3%)	495 (9.7%)
	**All-cause death**	131 (26.4%)	296 (15.8%)	171 (10.2%)	56 (6.6%)	5 (2.2%)	659 (12.9%)

Footnote/abbreviations: Participants are stratified by baseline eGFR categories(chronic kidney disease) stage (G1-G5), given as eGFR (estimated glomerular filtration rate in mL/min/1.73 m^2). eGFR slope rates are given in mL/min/1.73m^2 per year. Negative values reflect decline. Rapid decline is defined as a decline of more than 5 mL/min/1.73m^2/year. In the event that the endpoint (EP) was reached (EP 30%, 40%, 57% eGFR decline), the corresponding time (in years) was reported after the baseline. Kidney death: death due to forgoing of dialysis. KF: kidney failure: kidney failure with replacement therapy or kidney death. Mean reported with standard deviation (±SD), median with 25% and 75% quartiles (interquartile range IQR: Q25, Q75) or as absolute and relative frequencies. The total participant number is based on the complete case dataset.

Table S6. Model characteristics.

	Independent variables	A) Model 1: eGFR Estimates ml/min/1.73m²	CI	P	B) Model 2: eGFR Estimates ml/min/1.73m²	CI	P
Fixed Effects	(Intercept)	49.37	48.89 – 49.86	**<0.0001**	87.33	84.39 – 90.27	**<0.0001**
	**Follow-up time [years]**	-1.38	-1.45 – -1.31	**<0.0001**	-1.10	-1.56 – -0.64	**<0.0001**
	**sex [female]**				0.71	-0.21 – 1.64	0.1308
	**Age at baseline (years)**				-0.61	-0.66 – -0.57	**<0.0001**
	**UACR (mg/g)**						
	<30 (Reference)				--	--	**--**
	[30,299]				-3.76	-4.85 – -2.67	**<0.0001**
	[300,3000]				-2.67	-3.88 – -1.45	**<0.0001**
	>3000				-7.80	-10.60 – -5.01	**<0.0001**
	**Disease etiology**						
	Hypertensive kidney disease (reference)				--	--	**--**
	Primary glomerular disease				4.38	2.87 – 5.90	**<0.0001**
	Diabetic kidney disease				-0.39	-1.88 – 1.10	0.6055
	ADPKD				-8.41	-10.94 – -5.89	**<0.0001**
	Other kidney disease				0.56	-0.62 – 1.74	0.3533
Interaction with follow-up time [years]	**Time × sex [female]**				0.11	-0.04 – 0.25	0.1416
	**Time × age (years)**				0.01	0.0016 – 0.0145	**0.0143**
	**Time × UACR (mg/g)**						
	<30 (Reference)				--	--	**--**
	[30,299]				-0.52	-0.69 – -0.36	**<0.0001**
	[300,3000]				-1.93	-2.12 – -1.73	**<0.0001**
	>3000				-3.46	-3.95 – -2.96	**<0.0001**
	**Time × disease etiology**						
	Hypertensive kidney disease (reference)				--	--	--
	Primary glomerular disease				0.21	-0.03 – 0.44	0.0816
	Diabetic kidney disease				-0.60	-0.84 – -0.37	**<0.0001**
	ADPKD				-2.41	-2.80 – -2.01	**<0.0001**
	Other kidney disease				0.06	-0.13 – 0.24	0.5507
	σ^2	55.81			55.62		
	τ_{00}	300.32 participant			237.83 participant		
	τ_{11}	4.78 participant×time			3.87 participant×time		
	ρ_{01}	-0.06 participant			-0.03 participant		
	ICC	0.86			0.84		
	N	5214 participant [cleaned dataset]			5122 participant [complete case dataset]		
	Observations	49991			49217		
	Marginal R^2 / Conditional R^2	0.018 / 0.865			0.180 / 0.866		

Footnote/abbreviations: (A) Reference Model 1 only includes follow-up time in years as independent variable, as well as a participant-specific random intercept and slope. (B) Model 2 additionally includes baseline characteristics (UACR: urinary albumin to creatinine ratio, sex, age, and disease etiology) as fixed effects and as interaction effects with the follow-up time as independent variables. Reference categories: male sex, UACR <30, Hypertensive kidney disease, theoretical age of zero years. Patients without baseline UACR are excluded from Model 2, baseline characteristics can be found in **Table S1**. ADPKD: autosomal dominant polycystic kidney disease. Random effects parameters: τ_{00}: variance parameter for patient-specific intercept, τ_{11}: variance of patient-specific slope, ρ_{01}: random effect correlation, ICC: intraclass-correlation coefficient, σ^2: residual variance, N: number of participants (number of random effects), Observations: number of provided eGFR values.

Table S7. Sensitivity analysis: Model characteristics using only data available at follow-up visits.

	Independent variables	A) Model 1: eGFR Estimates ml/min/1.73m²	CI	P	B) Model 2: eGFR Estimates ml/min/1.73m²	CI	P
Fixed Effects	(Intercept)	48.71	48.23 – 49.20	**<0.0001**	84.77	81.82 – 87.73	**<0.0001**
	**Follow-up time [years]**	-1.37	-1.44 – -1.29	**<0.0001**	-1.18	-1.66 – -0.71	**<0.0001**
	**sex [female]**				1.16	0.23 – 2.09	**0.0144**
	**Age at baseline (years)**				-0.59	-0.63 – -0.55	**<0.0001**
	**UACR (mg/g)**						
	<30 (Reference)				--	--	**--**
	[30,299]				-3.47	-4.57 – -2.38	**<0.0001**
	[300,3000]				-2.05	-3.27 – -0.84	**0.00010**
	>3000				-5.75	-8.55 – -2.94	**0.0001**
	**Disease etiology**						
	Hypertensive kidney disease (reference)				--	--	**--**
	Primary glomerular disease				3.81	2.29 – 5.33	**<0.0001**
	Diabetic kidney disease				-0.45	-1.95 – 1.05	0.5541
	ADPKD				-8.46	-10.99 – -5.93	**<0.0001**
	Other kidney disease				0.37	-0.82 – 1.56	0.5430
Interaction with follow-up time [years]	**Time × sex [female]**				-0.06	-0.21 – 0.09	0.4023
	**Time × age (years)**				0.01	0.0022 – 0.0156	**0.0091**
	**Time × UACR (mg/g)**						
	<30 (Reference)				--	--	**--**
	[30,299]				-0.58	-0.75 – -0.40	**<0.0001**
	[300,3000]				-1.85	-2.05 – -1.65	**<0.0001**
	>3000				-3.04	-3.60 – -2.49	**<0.0001**
	**Time × disease etiology**						
	Hypertensive kidney disease (reference)				--	--	--
	Primary glomerular disease				0.28	0.04 – 0.52	0.0234
	Diabetic kidney disease				-0.49	-0.74 – -0.23	**0.0002**
	ADPKD				-2.04	-2.45 – -1.63	**<0.0001**
	Other kidney disease				0.14	-0.05 – 0.33	0.1481
	σ^2	44.99			43.95		
	τ_{00}	283.19 participant			226.64 participant		
	τ_{11}	2.84 participant×time			2.20 participant×time		
	ρ_{01}	-0.09 participant			-0.05 participant		
	ICC	0.87			0.85		
	N	5208 participant [cleaned dataset, only FU visits]			5116 participant [complete case dataset, only FU visits]		
	Observations	15186			14939		
	Marginal R^2 / Conditional R^2	0.025 / 0.873			0.188 / 0.876		

Footnote/abbreviations: Same models as in **Table S6**, but refitted only on available data from follow-up visits. Of note: Model fits with fewer observations resulted in convergence warnings in default settings and were therefore fitted using Nelder Mead optimizer. ADPKD: autosomal dominant polycystic kidney disease. Random effects parameters: τ_{00}: variance parameter for patient-specific intercept, τ_{11}: variance of patient-specific slope, ρ_{01}: random effect correlation, ICC: intraclass-correlation coefficient, σ^2: residual variance, N: number of participants (number of random effects), Observations: number of provided eGFR values.

Table S8. Model characteristics of interaction models.

		(A) ADPKD			(B) Diabetic kidney disease			(C) Primary glomerular disease			(D) Hypertensive kidney disease			(E) MISC		
	**Independent variables**	**Est.** ml/min/ 1.73m^2	**CI**	**P**	**Est.** ml/min/ 1.73m^2	**CI**	**P**	**Est.** ml/min/ 1.73m^2	**CI**	**P**	**Est.** ml/min/ 1.73m^2	**CI**	**P**	**Est.** ml/min/ 1.73m^2	**CI**	**P**
**Fixed Effects**	(Intercept)	45.48	42.41 – 48.55	**<0.0001**	48.51	46.23 – 50.79	**<0.0001**	59.26	57.12 – 61.41	**<0.0001**	50.18	48.52 – 51.84	**<0.0001**	52.96	51.52 – 54.40	**<0.0001**
	sex [female]	-0.20	-4.54 – 4.14	0.9282	0.34	-3.55 – 4.23	0.8633	5.26	1.72 – 8.81	**0.0036**	1.11	-1.70 – 3.92	0.4401	5.35	3.34 – 7.36	**<0.0001**
	age ≥ 63	-4.59	-10.30 – 1.12	0.1154	-4.86	-7.59 – -2.13	**0.0005**	-13.81	-17.68 – -9.94	**<0.0001**	-5.21	-7.21 – -3.22	**<0.0001**	-8.70	-10.63 – -6.76	**<0.0001**
	sex [f] × age ≥ 63	-2.92	-11.84 – 6.00	0.5209	-0.10	-4.85 – 4.64	0.9660	-4.59	-11.30 – 2.13	0.1805	-2.42	-5.79 – 0.95	0.1600	-5.30	-8.10 – -2.51	**0.0002**
**Interaction with follow-up time**	Time	-3.99	-4.53 – -3.46	**<0.0001**	-2.67	-3.11 – -2.22	**<0.0001**	-2.06	-2.36 – -1.77	**<0.0001**	-0.93	-1.22 – -0.64	**<0.0001**	-1.38	-1.61 – -1.16	**<0.0001**
	Time × sex [female]	-0.28	-1.03 – 0.48	0.4691	1.03	0.29 – 1.76	**0.0061**	0.19	-0.29 – 0.68	0.4335	0.04	-0.44 – 0.52	0.8765	0.32	0.01 – 0.63	**0.0420**
	Time × age ≥ 63	2.02	1.04 – 2.99	**<0.0001**	0.95	0.42 – 1.49	**0.0005**	0.74	0.21 – 1.28	**0.0065**	-0.15	-0.50 – 0.20	0.3899	0.26	-0.04 – 0.56	0.0943
	Time × sex [f] × age≥ 63	0.41	-1.11 – 1.94	0.5934	-0.74	-1.64 – 0.16	0.1064	-0.00	-0.91 – 0.91	0.9948	0.35	-0.23 – 0.94	0.2355	-0.02	-0.46 – 0.41	0.9144
**Random Effects**	σ^2	24.85			55.32			67.93			50.06			56.24		
	τ_{00}	165.39 participant			195.70 participant			497.99 participant			151.06 participant			238.26 participant		
	τ_{11}	3.86 participant×time			4.88 participant×time			6.93 participant×time			3.15 participant×time			3.91 participant×time		
	ρ_{01}	0.04 participant			-0.13 participant			-0.00 participant			-0.04 participant			-0.03 participant		
	ICC	0.90			0.81			0.90			0.79			0.84		
	N	191 participant			783 participant			977 participant			1198 participant			2065 participant		
	Observations	1739			7252			9333			11480			20187		
	Marginal R^2 / Conditional R^2	0.177 / 0.917			0.055 / 0.819			0.079 / 0.905			0.046 / 0.800			0.101 / 0.852		

Footnote/abbreviations: eGFR in mL/min/1.73m^2: dependent variable. Each model for interaction, Model (3), was fitted for each disease etiology separately. Reference: male sex, age younger than 63 years. Est.: Model estimate. Time: in years. MISC: other kidney disease. ADPKD: autosomal dominant kidney disease. Random effects parameters: τ_{00}: variance parameter for patient-specific intercept, τ_{11}: variance of patient-specific slope, ρ_{01}: random effect correlation, ICC: intraclass-correlation coefficient, σ^2: residual variance, N: number of participants (number of random effects), Observations: number of provided eGFR values.

Table S9. Number of participants reaching kidney related endpoints during 6.5 years of follow-up.

	**Male** (N=3131)	**Female** (N=2083)	**Overall** (N=5214)
**Kidney death**	19 (0.6%)	6 (0.3%)	25 (0.5%)
**KFRT**	355 (11.3%)	132 (6.3%)	487 (9.3%)
**All-cause death**	508 (16.2%)	171 (8.2%)	679 (13.0%)

Footnote/abbreviations: Participants are stratified by sex. Kidney death: death due to forgoing of dialysis.. KFRT: kidney failure treated by kidney replacement therapy .The total particpant number is based on the cleaned dataset.

Table S10. Model-based eGFR slopes, GFR related and other endpoints stratified by baseline UACR categories.

		**<30 mg/g** (N=2188)	**[30,299] mg/g** (N=1491)	**[300,3000] mg/g** (N=1297)	**>3000 mg/g** (N=146)	**Overall** (N=5122)
**Model (2) slope**	**eGFR slope**					
	Mean (SD)	-0.66 (1.39)	-1.29 (1.62)	-2.60 (1.92)	-4.09 (2.30)	-1.43 (1.86)
	Median [Q25, Q75]	-0.60 [-1.40, 0.11]	-1.14 [-2.06, -0.42]	-2.53 [-3.51, -1.51]	-4.44 [-5.46, -2.80]	-1.18 [-2.41, -0.31]
	Rapid decline	15 (0.7%)	33 (2.2%)	119 (9.2%)	54 (37.0%)	221 (4.3%)
**Estimated EPs derived from Model (2)**	**Absolut and relative frequency reaching estimated EP:** n (%)					
	**30% eGFR**	331 (15.1%)	425 (28.5%)	741 (57.1%)	116 (79.5%)	1613 (31.5%)
	**40% eGFR**	172 (7.9%)	267 (17.9%)	555 (42.8%)	107 (73.3%)	1101 (21.5%)
	**57% eGFR**	60 (2.7%)	127 (8.5%)	329 (25.4%)	87 (59.6%)	603 (11.8%)
	Missing	0 (0%)	2 (0.1%)	0 (0%)	0 (0%)	2 (0.0%)
	**Time EP reached:** Median [Q25, Q75]					
	**30% eGFR**	4.23 [2.95, 5.52]	4.07 [2.75, 5.24]	3.57 [2.40, 4.84]	2.13 [1.08, 3.22]	3.73 [2.47, 5.03]
	**40% eGFR**	4.62 [3.65, 5.53]	4.50 [3.19, 5.50]	4.14 [3.03, 5.20]	2.85 [1.77, 4.10]	4.22 [3.02, 5.25]
	**57% eGFR**	5.25 [4.54, 5.87]	5.02 [3.86, 5.76]	4.86 [3.79, 5.65]	3.88 [2.70, 4.66]	4.76 [3.66, 5.65]
**Observed EPs**	**Kidney death**	7 (0.3%)	9 (0.6%)	4 (0.3%)	4 (2.7%)	24 (0.5%)
	**KFRT**	60 (2.7%)	130 (8.7%)	227 (17.5%)	64 (43.8%)	481 (9.4%)
	**All-cause deaath**	254 (11.6%)	206 (13.8%)	171 (13.2%)	28 (19.2%)	659 (12.9%)

Footnote/abbreviations: Participants are stratified by baseline CKD (chronic kidney disease) stage (G1-G5), given as eGFR (estimated glomerular filtration rate in mL/min/1.73 m^2). The eGFR slopes are given in mL/min/1.73m^2/year. Negative values reflect decline. Rapid decline is defined as decline of more than 5 mL/min/1.73m^2/year. If the endpoint (EP) was reached (EP 30%, 40%, 57% eGFR decline), corresponding time (after baseline in years) was reported. The subpopulation with existing UACR baseline values (N=5122) as used in Model 2 is shown. Kidney death: death due to forgoing of dialysis. kidney failure: kidney failure with replacement therapy or kidney death. Mean reported with standard deviation (±SD), median with 25% and 75% quartiles (IQR: Q25, Q75) or as absolute and relative frequencies. The total participant number is based on the complete case dataset. KFRT: KF treated by kidney replacement therapy

Note S1. Current GCKD Investigators and Collaborators with the GCKD Study are:

University of Erlangen: Kai-Uwe Eckardt, Heike Meiselbach, Markus P. Schneider, Mario Schiffer, Hans-Ulrich Prokosch, Barbara Bärthlein, Andreas Beck, André Reis, Arif B. Ekici, Susanne Becker, Ulrike Alberth-Schmidt, Anke Weigel, Sabine Marschall, Eugenia Schefler;

University of Freiburg: Gerd Walz, Anna Köttgen, Ulla T. Schultheiß, Fruzsina Kotsis, Simone Meder, Erna Mitsch, Ursula Reinhard;

RWTH Aachen University: Jürgen Floege, Turgay Saritas, Alice Groß;

Charité, University Medicine Berlin: Elke Schaeffner, Seema Baid-Agrawal, Kerstin Theisen;

Hannover Medical School: Kai Schmidt-Ott;

University of Heidelberg: Martin Zeier, Claudia Sommerer, Mehtap Aykac;

University of Jena: Gunter Wolf, Martin Busch, Andy Steiner;

Ludwig-Maximilians University of München: Thomas Sitter;

University of Würzburg: Christoph Wanner, Vera Krane, Antje Börner-Klein, Britta Bauer;

Medical University of Innsbruck, Division of Genetic Epidemiology: Florian Kronenberg, Julia Raschenberger, Barbara Kollerits, Lukas Forer, Sebastian Schönherr, Hansi Weissensteiner;

University of Regensburg, Institute of Functional Genomics: Peter Oefner, Wolfram Gronwald;

Institute of Medical Biometry, Informatics and Epidemiology, Medical Faculty, University of Bonn: Matthias Schmid, Jennifer Nadal.

Supplementary References

S1. Prokosch HU, Mate S, Christoph J*, et al.* Designing and implementing a biobanking IT framework for multiple research scenarios. *Stud Health Technol Inform* 2012; **180:** 559-563.

S2. Levey AS, Stevens LA, Schmid CH*, et al.* A new equation to estimate glomerular filtration rate. *Ann Intern Med* 2009; **150:** 604-612.

S3. Pattaro CF. R. nephro: Utilities for Nephrology. 2022.

STROBE Statement

	Item No.	Recommendation	Page No.	Relevant text from manuscript
**Title and abstract**	1	(*a*) Indicate the study's design with a commonly used term in the title or the abstract	1, 2	CKD cohort study
		(*b*) Provide in the abstract an informative and balanced summary of what was done and what was found	2	In the German CKD study involving 5,214 participants, eGFR slopes were modeled over 6.5 years using linear-mixed effects models to assess heterogeneity and associations with CKD etiology, eGFR/UACR, age, sex, and outcomes, revealing an average annual slope of -1.43 mL/min/1.73m² that varied significantly by UACR, etiology, and age, with faster declines in younger participants and those with polycystic or diabetic kidney disease.
**Introduction**				
Background/rationale	2	Explain the scientific background and rationale for the investigation being reported	3, 4	This study was conducted to address the need for a deeper understanding of chronic kidney disease (CKD) heterogeneity by analyzing eGFR slopes as a surrogate marker of CKD progression across a broad spectrum of patients, leveraging data from over 5,000 participants in the German CKD study to explore associations with CKD classification, participant characteristics, and clinical outcomes.
Objectives	3	State specific objectives, including any prespecified hypotheses	4	The objective was to analyze eGFR slope heterogeneity and its associations with CKD classification, patient characteristics, and clinical outcomes in a large CKD cohort.
**Methods**				
Study design	4	Present key elements of study design early in the paper	6	Over a follow-up of 6.5 years, eGFR slopes were modeled using linear mixed-effects models, excluding data affected by AKI or KFRT, and stratified by quintiles, CKD categories, and demographic factors to analyze heterogeneity and associations with estimated (30%, 40%, 57% eGFR decline) and observed endpoints (KFRT, kidney death, all-cause mortality).
Setting	5	Describe the setting, locations, and relevant dates, including periods of recruitment, exposure, follow-up, and data collection	5, 6, 7	The study was conducted within the German CKD (GCKD) cohort, enrolling 5,217 participants from nephrology practices across Germany between 2010 and 2012. Participants were followed for up to 6.5 years, with data collection including per-protocol and routine clinical measurements of eGFR and UACR over the follow-up period. The dataset used for analysis included information up to the data freeze in March 2022.
Participants	6	(*a*) *Cohort study*—Give the eligibility criteria, and the sources and methods of selection of participants. Describe methods of follow-up	5, 6, 7	Participants were eligible if they met one of the following criteria:<br>1. eGFR between 30–60 mL/min/1.73m² (CKD G3, A1-3; 91.6% of participants).<br>2. eGFR >60 mL/min/1.73m² with overt albuminuria, defined as a UACR ≥300 mg/g (CKD G1-2, A3; 8.4% of participants).

		Case-control study—Give the eligibility criteria, and the sources and methods of case ascertainment and control selection. Give the rationale for the choice of cases and controls<br><br>*Cross-sectional study*—Give the eligibility criteria, and the sources and methods of selection of participants		Exclusion criteria included: Individuals with non-German speaking ability or inability to provide informed consent.<br><br>Participants were recruited from nephrology practices across Germany between 2010 and 2012. Selection was based on baseline measurements of eGFR and UACR, and participants were categorized into KDIGO risk groups (low, moderate, high, and very high) based on these parameters.<br><br>Participants were followed for up to 6.5 years, during which eGFR values were collected from:<br><br>1. Per-protocol measurements performed at scheduled study visits.<br>2. Routine clinical data from medical records.<br>Acute kidney injury (AKI) events were adjudicated by trained physicians using discharge reports and categorized using KDIGO definitions. Any eGFR measurements taken after kidney failure requiring replacement therapy (KFRT) or during AKI (±7 days from the acute event) were excluded. Data collection and cleaning were completed by March 2022.
		(*b*) *Cohort study*—For matched studies, give matching criteria and number of exposed and unexposed<br><br>*Case-control study*—For matched studies, give matching criteria and the number of controls per case		
Variables	7	Clearly define all outcomes, exposures, predictors, potential confounders, and effect modifiers. Give diagnostic criteria, if applicable	5, 6, 7 Supplement page 3	Outcomes: Estimated Endpoints: Relative eGFR declines of 30%, 40%, and 57% from baseline, calculated using modeled eGFR trajectories.<br><br>Observed Endpoints: Kidney failure requiring replacement therapy (KFRT: dialysis or transplantation); Kidney death (death due to forgoing dialysis); All-cause mortality.<br><br>Exposure: Time (follow-up period): Used in models to calculate eGFR slopes, with changes in eGFR over time serving as the primary marker of CKD progression.<br><br>Predictors: Baseline characteristics (Demographics: Age, sex; Clinical measures: eGFR, UACR; CKD etiology: Polycystic kidney disease (ADPKD), diabetic kidney disease (DKD), primary glomerular disease (PGD), hypertensive kidney disease (HKD), and other kidney diseases (MISC)<br><br>Interaction effects: Age categories (younger vs. older than median); Sex (male vs. female).<br><br>Confounders: Variables that could influence both eGFR slope and outcomes (Baseline eGFR and UACR levels; CKD etiology; Age and sex)<br><br>Effect Modifiers

				Variables that could modify the relationship between eGFR slope and outcomes (CKD etiology: Differences in progression patterns by disease type; Age: Younger participants tended to have faster eGFR decline; Sex: Interaction effects, particularly in participants with diabetic kidney disease, where women had lower eGFR slopes than men; UACR: Faster declines observed with higher levels of albuminuria.)
Data sources/ measurement	8*	For each variable of interest, give sources of data and details of methods of assessment (measurement). Describe comparability of assessment methods if there is more than one group	5, 6, 7 Supplement page 3	Data sources included structured study visits, routine clinical care records, and medical documentation. Key variables were assessed as follows: eGFR was calculated using the CKD-EPI equation based on serum creatinine measurements from study visits and clinical care, excluding values after KFRT or during AKI (adjudicated using KDIGO criteria). UACR was measured from urine samples to assess albuminuria. CKD etiology was determined by nephrologists at baseline, categorized into ADPKD, DKD, PGD, HKD, or MISC. Age and sex were recorded at baseline. Outcomes included KFRT (dialysis or transplantation), mortality (all-cause and kidney-specific, determined from death certificates and records), and estimated eGFR declines (30%, 40%, 57%) based on linear mixed-effects models. Interaction terms (e.g., age, sex, UACR) were included to explore their influence on eGFR slope.
Bias	9	Describe any efforts to address potential sources of bias	5, 6, 7, 18 Supplement page 3	Efforts to address potential sources of bias included excluding eGFR values during AKI or after KFRT, using linear mixed-effects models to account for repeated measurements and missing data, and adjusting for confounders like age, sex, UACR, and CKD etiology. Stratification by CKD categories and interaction terms helped identify modifying effects, while sensitivity analyses using follow-up data further validated the findings. Physician adjudication of AKI ensured consistent event classification. Limitations of the study, including potential biases, are discussed in the discussion section.
Study size	10	Explain how the study size was arrived at	6, Supplement Flow chart page 6	The cleaned data set comprised data from 5,214 participants for a period of up to 6.5 years following the baseline measurement. Three participants were excluded due to missingness in eGFR values.
Quantitative variables	11	Explain how quantitative variables were handled in the analyses. If applicable, describe which groupings were chosen and why	5, 6, 7	The handling of quantitative variables like eGFR involved complex statistical modeling to account for repeated measures, covariates, interactions, and subgroup differences. The use of linear mixed-effects models allowed for individual-specific estimations of eGFR slope, and stratification into quintiles helped to better understand the heterogeneity of eGFR decline across the study population. These analyses facilitated the assessment of eGFR trajectories in relation to clinical outcomes and provided a detailed description of kidney function changes over time.
Statistical methods	12	(*a*) Describe all statistical methods, including those used to control for confounding	6, 7	The statistical methods combined linear mixed-effects models for handling repeated measures, covariate adjustments, and subgroup stratification to account for confounding variables like sex, age, and disease etiology. Sensitivity analyses and exclusion of participants with missing baseline data

				further enhanced the robustness of the results. The use of interaction terms and stratification allowed for a nuanced understanding of how different factors affected eGFR decline over time.
		(*b*) Describe any methods used to examine subgroups and interactions	6	Subgroup analyses were conducted by stratifying participants based on key characteristics such as **disease etiology, eGFR categories (G1-G5),** and **albuminuria levels (A1-A3)** to explore how these factors influenced eGFR decline. Additionally, interaction effects between **sex** and **age** (older vs. younger than the median) were tested separately for each disease etiology to assess how these demographic factors modified the relationship between eGFR decline and follow-up time. These subgroup and interaction analyses helped identify differences in eGFR slopes across distinct participant groups, providing a deeper understanding of how baseline factors might influence kidney function decline over time.
		(*c*) Explain how missing data were addressed	6	Participants with **missing baseline eGFR** data were excluded from the analysis
		(*d*) *Cohort study*—If applicable, explain how loss to follow-up was addressed<br><br>*Case-control study*—If applicable, explain how matching of cases and controls was addressed<br><br>*Cross-sectional study*—If applicable, describe analytical methods taking account of sampling strategy	6	Loss to follow-up was addressed by using **linear mixed-effects models**, which can handle incomplete data by modeling repeated measurements over time, even for participants who missed some follow-up visits.
		(*e*) Describe any sensitivity analyses	6	Sensitivity analyses were carried out using data from follow-up visits only, excluding baseline measurements. This was done to determine whether the inclusion of baseline data affected the results, providing insight into the robustness of the findings when focusing strictly on changes in eGFR over time.
Results				
Participants	13*	(a) Report numbers of individuals at each stage of study—eg numbers potentially eligible, examined for eligibility, confirmed eligible, included in the study, completing follow-up, and analysed	8, Supplement Flow chart page 6	A total of **5,217** participants were eligible for the study. After data cleaning, **5,214** participants were confirmed. These participants were followed for up to 6.5 years, with **49,991 eGFR values** collected for analysis. The data were derived from a combination of in-person follow-up visits (30%) and medical reports (70%), with a median of 9 eGFR values per participant. Ultimately, the study analyzed data from **5,214** participants, ensuring a comprehensive dataset for the research.
		(b) Give reasons for non-participation at each stage	Supplement flow chart page 6	Initially, some participants were excluded from the study due to missing **baseline eGFR data** or other incomplete baseline measurements, resulting in the exclusion of **3 participants** who lacked any eGFR values. During data cleaning, additional exclusions occurred for **eGFR values obtained after kidney failure or renal replacement therapy (KFRT),** with

			**4,076 eGFR values** excluded. Similarly, **2,530 eGFR values** were excluded due to acute kidney injury (AKI) events. Participants were also censored after **6.5 years** of follow-up, leading to the removal of **19,732 eGFR values.**
		(c) Consider use of a flow diagram	See page 6 supplement
Descriptive data	14*	(a) Give characteristics of study participants (eg demographic, clinical, social) and information on exposures and potential confounders	8, 9	The study participants varied in age, sex, kidney disease etiology, and baseline eGFR and UACR, with key confounders including age, sex, disease etiology, and UACR, all considered in the analyses to assess eGFR decline.
		(b) Indicate number of participants with missing data for each variable of interest		See page 6 supplement flow chart
		(c) *Cohort study*—Summarise follow-up time (eg, average and total amount)	6	Follow-up time was 6.5 years (Q1:6.5, Q3:6.5)
Outcome data	15*	*Cohort study*—Report numbers of outcome events or summary measures over time	*9-11*	Over the study period, **487 participants** (9.3%) experienced kidney failure requiring renal replacement therapy (KFRT). A total of **679 participants** (13.0%) died during the follow-up, with **25 participants** (0.5%) experiencing kidney death due to forgoing kidney replacement therapy. Mortality was higher in men, and participants in the "fast decline" quintile had more than double the mortality rate compared to those in the "slow decline" quintile (19.6% vs. 8.1%). Additionally, a greater proportion of participants with **UACR >3000 mg/g** reached eGFR endpoints (e.g., 40% decline) in a shorter time compared to those with **UACR <30 mg/g.**
		Case-control study—Report numbers in each exposure category, or summary measures of exposure		
		Cross-sectional study—Report numbers of outcome events or summary measures		
Main results	16	(*a*) Give unadjusted estimates and, if applicable, confounder-adjusted estimates and their precision (eg, 95% confidence interval). Make clear which confounders were adjusted for and why they were included	6, 9, Tables 1, 2, S5, S6	In an adjusted Model 2, the baseline variables UACR, sex, age, and disease etiology were additionally included as fixed effects and as interaction effects with the follow-up time using a complete case dataset. Unadjusted estimates of eGFR slopes showed an overall mean annual decline of **-1.38 mL/min/1.73m².** After adjusting for confounders in Model 2, the mean annual eGFR slope was **-1.43 mL/min/1.73m²,** with estimates ranging from **-4.19** (fastest decline) to **+0.85** (slowest decline).

	(*b*) Report category boundaries when continuous variables were categorized		6, 9, 10	Continuous variables were categorized based on clinically relevant thresholds (G/A stages for eGFR/UACR).
	(*c*) If relevant, consider translating estimates of relative risk into absolute risk for a meaningful time period		9, 10	Relative risk estimates, such as the likelihood of a 40% eGFR decline, can be translated into absolute risk by considering the proportion of participants affected within each subgroup. For example, in the ADPKD group, 65.8% experienced a 40% eGFR decline over 3.6 years, and participants with UACR >3000 mg/g reached eGFR decline thresholds more quickly, emphasizing the role of UACR in predicting risk.
Other analyses	17	Report other analyses done—eg analyses of subgroups and interactions, and sensitivity analyses	10-13	Differentiation of eGFR slopes by age, sex, and disease etiology and comparison of eGFR slopes and endpoints by disease etiology and UACR.
**Discussion**				
Key results	18	Summarise key results with reference to study objectives	14	The study found that CKD progression varied widely across disease etiologies, with ADPKD showing the fastest eGFR decline. UACR was strongly linked to progression, and younger males with higher baseline UACR had faster declines. These findings highlight the value of using individual eGFR slopes to refine CKD management.
Limitations	19	Discuss limitations of the study, taking into account sources of potential bias or imprecision. Discuss both direction and magnitude of any potential bias	18	The study has limitations, including a cohort limited to nephrology patients, Caucasian ethnicity, and reliance on both central and routine eGFR data, which may introduce variability. eGFR equations may overestimate slopes in younger patients and underestimate in older ones.
Interpretation	20	Give a cautious overall interpretation of results considering objectives, limitations, multiplicity of analyses, results from similar studies, and	14-17	The study provides valuable insights into CKD progression, highlighting disease heterogeneity and the role of etiology and UACR in eGFR decline. Findings align with previous studies but should be interpreted cautiously due to the multiplicity of analyses and study design. Further research is needed to refine individualized management strategies.

		other relevant evidence		
Generalisability	21	Discuss the generalisability (external validity) of the study results	18	The study's generalisability is limited by its focus on nephrology-referred, predominantly Caucasian patients, which may not reflect the broader CKD population. Strict inclusion criteria and variability in eGFR measurements also affect generalizability, though the modeling approach helps address this. Despite these limitations, the findings provide useful insights for similar patient populations.
**Other information**				
Funding	22	Give the source of funding and the role of the funders for the present study and, if applicable, for the original study on which the present article is based	19	All funding is listed.

*Give information separately for cases and controls in case-control studies and, if applicable, for exposed and unexposed groups in cohort and cross-sectional studies.

Note: An Explanation and Elaboration article discusses each checklist item and gives methodological background and published examples of transparent reporting. The STROBE checklist is best used in conjunction with this article (freely available on the Web sites of PLoS Medicine at http://www.plosmedicine.org/, Annals of Internal Medicine at http://www.annals.org/, and Epidemiology at http://www.epidem.com/). Information on the STROBE Initiative is available at www.strobe-statement.org.
